# TSTBench: A Comprehensive Benchmark for Text Style Transfer

**DOI:** 10.3390/e27060575

**Published:** 2025-05-29

**Authors:** Yifei Xie, Jiaping Gui, Zhengping Che, Leqian Zhu, Yahao Hu, Zhisong Pan

**Affiliations:** 1 Command and Control Engineering College, Army Engineering University, Nanjing 210007, China; 2 School of Computer Science, Shanghai Jiaotong University, Shanghai 200240, China; 3 Beijing Innovation Center of Humanoid Robotics, Beijing 100176, China

**Keywords:** text style transfer, large language models (LLMs), text generation, transformer, benchmark, deep learning, natural language processing (NLP)

## Abstract

In recent years, researchers in computational linguistics have shown a growing interest in the style of text, with a specific focus on the text style transfer (TST) task. While numerous innovative methods have been proposed, it has been observed that the existing evaluations are insufficient to validate the claims and precisely measure the performance. This challenge primarily stems from rapid advancements and diverse settings of these methods, with the associated (re)implementation and reproducibility hurdles. To bridge this gap, we introduce a comprehensive benchmark for TST known as **TSTBench**. TSTBench includes a codebase encompassing implementations of 13 state-of-the-art algorithms and a standardized protocol for text style transfer. Based on the codebase and protocol, we have conducted thorough experiments across seven datasets, resulting in a total of 7000+ evaluations. Our work provides extensive analysis from various perspectives, explores the performance of representative baselines across various datasets, and offers insights into the task and evaluation processes to guide future research in TST.

## 1. Introduction

As an important task in natural language processing, text style transfer (TST) aims to automatically change the style (e.g., emotion, formality, politeness, genre, and syntax) of text while preserving the main semantic content. This task is inherently linked to concepts in information theory, such as information preservation and entropy, as it involves transforming stylistic features without compromising the underlying meaning of the text. TST can be widely applied to natural language generation in many scenarios, such as human–machine dialogue, text formalization, translation into specific styles, and poetry generation. Despite the progress in new methods, TST lags behind other subfields of AI, such as computer vision with respect to style transfer. This situation is caused by various factors. Most research [1] has focused on learning style and content representations of text in an unsupervised manner due to the lack of parallel corpus. Another crucial reason is that we lack of a standardized criterion for evaluating these new methods. A recent study [2] calls for experimental comparisons to rank existing methods based on unified metrics. This situation is reflected in multiple aspects (e.g., there exist several commonly used methods for evaluating content preservation such as self-BLEU, ref-BLEU, BERT, BLEURT, etc.), which we explain in more detail below.

**(1) Utilization of Different Datasets**: The use of different datasets poses a challenge for comparing the performance of new methods. Even when using the same dataset, differences in format (e.g., whether pre-processed or not) can compromise the quality of model generation, ultimately impacting the model output [3]. Some methods may inherently only cater to some specific style or only one direction of style transfer, while others neglect to specify the direction altogether (e.g., [4,5,6]).

**(2) Diverse Mechanisms for Model Inputs**: New methods employ various mechanisms to generate model inputs. Most rely on pretrained language models (PLMs) to generate text representations (e.g., [7,8,9]), whereas others rely directly on task datasets (e.g., [4,5,6]). When conducting comparisons, it is more reasonable to specify whether a pre-trained model was used.

**(3) Variation in Evaluation Metrics**: New methods are often insufficiently evaluated, with varying evaluation setups, as shown in Table A1 in the Appendix A. Even for identical model output, the results of one metric, such as accuracy, may diverge from another based on different classification approaches. Furthermore, applying the same evaluation metrics to datasets with distinct styles has limitations due to differing levels of difficulty and unique characteristics within each task.

**(4) Unfair Evaluation**: Various factors, including diverse implementation settings (e.g., hyper-parameters for training) could potentially impact fair evaluations. These factors ought to be meticulously controlled when comparing TST methods, akin to how they are controlled in an ablation study for a single method.

Therefore, it is crucial to evaluate new methods using the same criteria. Otherwise, it becomes challenging to determine whether a new method outperforms its counterparts based on its foundational assumptions. Unfortunately, to the best of our knowledge, the current benchmarks do not adequately support thorough comparisons among methods for TST, as illustrated in Table 1. To bridge this gap, we have developed a comprehensive benchmark for TST, called **TSTBench**. The framework is built on an extensible modular-based codebase, comprising an input module, a transfer module, and an evaluation and analysis module. To ensure fair and reproducible evaluations, we provide a standardized protocol covering every step of the transfer process. Currently, we have incorporated 13 SOTA methods on TST and multiple visual analysis tools; researchers can easily integrate new methods and datasets into the evaluation framework for fair comparison. In this work, we also present the analysis from three perspectives: the effect of algorithms and datasets, the comparison between large language models (LLMs) and fine-tuned models, and the correlation between human and evaluation metrics, respectively. We summarize our contributions as follows.

- We replicate and compare 13 text style transfer algorithms across seven datasets, providing both code and outputs.
- We provide a unified evaluation framework, utilizing 10 different evaluation metrics, which yield over 7000 evaluation outcomes. Our modular architecture allows researchers to integrate new methods and datasets, facilitating fair comparisons within a standardized evaluation environment.
- We conduct a thorough analysis and have obtained new findings and conclusions. We have made our code publicly available on GitHub (https://github.com/FayeXXX/A-Benchmark-of-Text-Style-Transfer (accessed on 28 April 2025)).

## 2. Related Work

### 2.1. TST Algorithms

Text style transfer (TST) has emerged as a significant area of research in natural language processing, aiming to transform text from one style to another while preserving its original content [15,16,17,18,19]. Early methods aim to learn and separate the content and style of text [20,21]. Many approaches adopt disentanglement strategies, mapping text into a latent space to obtain latent representations that separate content and attributes, achieving some promising progress [20,21,22,23,24,25,26]. Subsequently, numerous strategies utilizing reinforcement learning emerged, designing various reward functions to enable models to better learn the representations of text content and style, as demonstrated in [27,28,29,30]. Other methods employ back-translation strategies, such as [31,32]. Additionally, some approaches use pseudo-parallel corpora for training, as seen in [33,34,35]. Further techniques focus on separating the content and style of text from the perspective of the text itself, using strategies that involve deleting and replacing style-related words [36,37,38,39,40,41].

To date, large-scale pre-trained language models (PLMs) like BERT [42], RoBERTa [43], XLNet [44], GPT [45], BART [46], ELECTRA [47], and T5 [48] have gradually become the new paradigm in natural language processing. Leveraging large corpora and unsupervised learning based on the transformer architecture, these models achieve state-of-the-art (SOTA) performance with merely fine-tuning on downstream tasks. As such, enhancing transformer-based models for text style transfer has become a popular research topic. This paper, therefore, focuses on transformer-based models. These methods can be categorized into three main groups based on their approach to parameter tuning during training: full-parameter fine-tuning, parameter-efficient fine-tuning, and zero-shot or few-shot prompting.

**(1) Full-Parameter Fine-Tuning (FPFT).** These methods can be further categorized into three groups depending on their approach to controlling text style [6]: embedding-based, decoder-based, and classifier-based. Embedding-based methods (e.g., CAAE [49], NAST [50], StyIns [24], TSST [6], and Styletransformer [4]) leverage style embeddings to direct the style of generated text. The decoder-based approach involves using dedicated decoders for each style transformation direction to control text style and includes Multi-decoder [51], STRAP [9], BSRR [8], TYB [7], and DualRL [52]. The classifier-based approaches, such as PPLM [53], Gradient-guided TST [54], RevisionTST [55], and CTAT [5], adjusts the latent representation of text through a well-trained style classifier.

**(2) Parameter-Efficient Fine-Tuning (PEFT).** These approaches aim to effectively fine-tune LLMs by training only a small proportion of parameters [56]. These parameters can either be a subset of the current model parameters (e.g., BitFit [57], DiffPruning [58]) or a newly added set of parameters (e.g., Adapter [59], prefix-tuning [60], prompt-tuning [61], LoRA [62]).

**(3) Zero-Shot or Few-Shot Prompting (ZSFS).** LLMs like ChatGPT [63], InstructGPT [64], PaLM [65], and GPT4 [66], have shown promising results in text genration. Meanwhile, CAT [67], ICLEF [68], and StyleChat [69] design prompting methods to Chatgpt and GPT4 for text style transfer on formality and authorship datasets. Each of these methods has its unique strengths and weaknesses; we select some representative algorithms to evaluate within our TSTBench.

### 2.2. Related Benchmarks

Several benchmarks have been introduced to evaluate TST tasks, such as GYAFC [10], MATST [11], CDS [9], StylePTB [12], XFORMAL [13], and LMStyle [14]. However, as shown in Table 1, these benchmarks often focus on a limited number of style types and evaluate only a subset of available algorithms. Additionally, they may lack comprehensive evaluation metrics, making it challenging to compare different approaches effectively. The work most similar to ours is the benchmark presented by Hu et al. [70]. This study is notable for its survey of TST methods but lacks detailed experimental settings and evaluations, which are crucial for standardizing benchmarks. Our work, TSTBench, addresses these limitations by providing a comprehensive evaluation of a wide range of TST algorithms across multiple style types, detailed experimental settings and configurations to ensure reproducibility, an extensive set of evaluation metrics to assess various aspects of TST performance, and in-depth analysis of the results.

## 3. TSTBench

We evaluate 13 TST algorithms across seven datasets using 10 different metrics, alongside human evaluations. In the subsequent subsections, we provide comprehensive details: Section 3.1 introduces the baseline algorithms, Section 3.2 discusses the datasets, Section 3.3 describes the evaluation metrics, and Section 3.4 offers an overview of the codebase.

### 3.1. Baseline Algorithms

We include 13 TST algorithms for evaluation in our benchmark, as shown in Table 2. Transformer-based models have dramatically improved performance across various NLP tasks and now represent the leading approach in the field. As a result, this paper focuses on algorithms that leverage the transformer architecture. These algorithms are either well-known or the state-of-the art, representing the latest advancements in the field. Specifically, seven algorithms (STYTRANS [4], TSST [6], NAST [50], STRAP [9], BSRR [8], TYB [7], and CTAT [5]) are classic methods that span three strategies of FPFT. The remaining six algorithms—LlaMa-LORA, LlaMa-LORA-INST [62], CHATGPT [63], CHATGPT-FS [63], GPT4 [66], and GPT4-FS [66]—are recently published methods based on LLMs. This selection allows us to compare the performance between fine-tuned models and traditional models versus LLMs. The details of the implemented algorithms are presented in Appendix A.1.

### 3.2. Datasets

We employ datasets representing both high-level and fine-grained styles, as detailed in Table 3. Our selection process is guided by two main criteria: First, the chosen datasets must be well-maintained and widely recognized, while also covering a diverse range of text types. Second, each dataset must contain a sufficient number of instances for experimentation. Ultimately, we focus on seven datasets that correspond to four types of TST tasks:

**(1) Sentiment Transfer.** We leverage two widely used sentiment datasets, Yelp [36] and Amazon [71]. Both datasets contain non-parallel data, derived from restaurant reviews on Yelp and product reviews on the Amazon website, respectively. For the Yelp test dataset, we utilize the data provided by [52], where each source-style sentence corresponds to four target-style sentences for reference.

**(2) Formality Transfer.** GYAFC (EM) or GYAFC (FR) [10] is the most commonly used formality dataset that originate from entertainment/music and family relationship-themed content, respectively. We utilize the test dataset according to the data in [10], incorporating four reference target-style sentences for each testing sentence.

**(3) Ancient English Transfer.** We leverage the Shakespeare dataset [72], which comprises both Shakespearean-style Old English and modern English data. This dataset presents a unique opportunity to explore style transfer across different linguistic and temporal contexts.

**(4) Syntactic/Semantic Style Transfer.** We also include the recently proposed StyPTB-TFU and StyPTB-ARR datasets [12]. The former focuses on syntactic style transfer that updates the present tense to the future tense in the text. The latter explores semantic style transfer, involving the removal of adjectives and adverbs from the text. Notice that we do not include other datasets under StyPTB because of their limited sample sizes, which range from two hundred to two thousand samples.

We follow the established practices for each model and partition the datasets into training, validation, and testing sets, as outlined in Table 3. This ensures consistency with prior research methodologies. Before initiating training, we pre-process the data by cleaning and truncating them. Specifically, we remove additional white spaces or punctuation marks separated by spaces within sentences [3]. Additionally, we truncate sentences to 64 words based on the average length.

### 3.3. Evaluation Metrics

TSTBench utilizes both a uniform set of automatic metrics and human evaluation to systematically evaluate different TST algorithms in terms of style strength, content preservation, and fluency. Below, we elaborate on evaluation methods in detail.

#### 3.3.1. Automatic Evaluation

The evaluation of automated metrics on text style transfer primarily encompasses three key characteristics: style strength, content preservation, and fluency.

**(1) Style strength**. Assessing the style strength of TST often involves training a binary classifier to determine if the generated sentences align with the target style [73]. This characteristic is typically measured by the accuracy metric, as the ACC (accuracy) of the output sentences reflects the success rate. Specifically, given input sentences $x_{i}$ in style *i* and the target style *j*, the classification model is trained to determine whether the output sentences $y_{j}$ adhere to style *j*. This includes utilizing various classifiers such as a linear classifier (e.g., fastText [74]), a 3-layer CNN (e.g., TextCNN [75]), and a classifier based on RoBERTa [43]. The training process employs cross-entropy loss to optimize the classification model. In information theory, cross-entropy measures the difference between two probability distributions—the true distribution of the data and the predicted distribution by the model. Minimizing cross-entropy loss effectively reduces the uncertainty in predictions, aligning with the goal of improving the model’s ability to accurately capture the stylistic properties of the target style. We leverage the RoBERTa classifer [43] due to its outstanding performance, as shown in Table 4. In our analysis, higher accuracy in evaluating the ground truth indicates superior performance of the respective evaluation model, highlighting its ability to accurately identify sentences that adhere to the desired style. However, using trainable classifiers can lead to biases during evaluation, particularly because these models, like many neural network models, can be susceptible to adversarial examples, as discussed in [1]. This issue is especially pronounced in sentiment classification tasks, where positive and negative sentences may differ by only a single word, making it challenging for RoBERTa to provide high-confidence judgments. In this work, we present the RoBERTa classifier as a baseline reference.

**(2) Content preservation.** BLEU stands as the most commonly employed metric and can be formulated as follows:

(1) $$BLEU=BP\times {\left(\prod_{n=1}^{4}P_{n}\right)}^{\frac{1}{4}},$$

where BP represents the brevity penalty and $P_{n}$ calculates the precision of n-grams between candidates *C* to be evaluated and a collection of references.

(2) $$P_{n}=\frac{\sum_{C\in \left\{\mathrm{Candidates}\right\}}\sum_{n-\mathrm{gram}\in C}{\mathrm{Count}}_{\mathrm{match}}\left(n-\mathrm{gram}\right)}{\sum_{C^{\prime }\in \left\{\mathrm{Candidates}\right\}}\sum_{{n-\mathrm{gram}}^{\prime }\in C^{\prime }}\mathrm{Count}\left({n-\mathrm{gram}}^{\prime }\right)},$$

where ${\mathrm{Count}}_{\mathrm{match}}\left(n-\mathrm{gram}\right)$ indicates the number of times an n-gram in the model’s output matches an n-gram in the reference sentence and $\mathrm{Count}({n-\mathrm{gram}}^{\prime })$ represents the total occurrences of each n-gram in the candidate sentences, ensuring precision considers both matched and unmatched elements.

The brevity penalty (BP) is applied to penalize translations that are shorter than their corresponding reference texts, adjusting the score as a corrective measure:

(3) $$BP=\left\{\begin{array}{cc} 1 & \;\mathrm{if}\;c>r\\ \exp\left(1-\frac{r}{c}\right) & \;\mathrm{if}\;c\leq r \end{array}\right..$$

Here, *c* represents the length of the candidate translation, and *r* is the length of the reference translation. This adjustment ensures that overly short translations do not achieve artificially high scores due to higher precision metrics.

However, these methods do not consider the inevitable alteration of style words during text style transfer [76] and are unable to determine the semantic similarity of synonyms. For example, in the sentiment transfer dataset, the original sentence “The food was tasteless and dry.” is transformed into “The food was flavorful and moist.”, resulting in a BLEU score of 0.3 despite most of the content being preserved. When the core content remains unchanged while only the style words are altered, BLEU tends to assign the low score. This occurs because BLEU’s n-gram matching mechanism fails to distinguish between style words and content words [1]. Most existing benchmarks did not consider these factors. A recent study by [77] shows that BERTScore metrics [78] can be utilized to a certain extent as a substitute for human evaluation. The language model enables the utilization of BERTScore metrics that harness their linguistic capabilities, thereby eliminating the sole dependence on superficial features of n-grams. The BERTScore calculates the precision and recall metrics by comparing each token representation *x* of the reference translation to each token representation $\hat{x}$ of the candidate translation. Specifically, $P_{\mathrm{BERT}}$ measures how well tokens in the candidate match those in the reference, while $R_{\mathrm{BERT}}$ assesses how well tokens in the reference are represented in the candidate. The harmonic mean of these scores is given by $F_{1}$, providing an overall measure of similarity:

(4) $$\begin{array}{c} P_{\mathrm{BERT}}=\frac{1}{\hat{\left|x\right|}}\sum_{{\hat{x}}_{j}\in \hat{x}}\max_{{\hat{x}}_{i}\in x}x_{i}^{⊤}{\hat{x}}_{j}\\ R_{\mathrm{BERT}}=\frac{1}{\left|x\right|}\sum_{x_{i}\in x}\max_{{\hat{x}}_{j}\in \hat{x}}x_{i}^{⊤}{\hat{x}}_{j}\\ F_{1}=2\frac{R_{\mathrm{BERT}}\cdot P_{\mathrm{BERT}}}{R_{\mathrm{BERT}}+P_{\mathrm{BERT}}}. \end{array}$$

To mitigate BLEU’s limitations, adjusting the weights in the BLEU calculation to assign higher importance to non-style n-gram matches or combining BLEU with the BERTScore as a hybrid metric could be an effective solutions We provide both BLEU and the BERTScore [78] to compare the output sentences with the input sentences (i.e., s-BLEU, s-BERT), or with reference sentences (i.e., r-BLEU, r-BERT; multi-BLEU, multi-BERT, if multiple references exist). We calculate the BLEU score using all available references in cases where human-written references exist.

**(3) Fluency.** Traditional methods to evaluate fluency often rely on manual human assessment in several aspects, including grammar, readability, and naturalness. However, to complement these subjective evaluations, Perplexity (PPL) offers an automated metric. PPL is defined as the exponentiated average negative log-likelihood of a sentence, given the language model LM, focusing on predicting the next word in a sequence based on training data:

(5) $$\mathrm{PPL}=\exp\left\{-\frac{1}{N}\sum_{i=1}^{N}\log\mathrm{LM}\left(w_{i}|w_{1:i-1}\right)\right\},$$

where $w_{i}$ denotes the *i*-th word in the sentence, and *N* is the total number of words in the sentence. PPL leverages the inherent capabilities of language models such as KenLM [79] and GPT-2 [80]. However, challenges remain regarding unbounded values and the observation that sentences with common words can yield low perplexity scores. To address this, [9] introduces a RoBERTa-large classifier trained on the Corpus of Linguistic Acceptability (CoLA) [81], a grammar-checking classifier used to assess grammatical acceptability [9].

PPL evaluates fluency based on a language model’s training distribution, which suffers from model bias and is style-agnostic, while CoLA serves as a binary grammatical norm checker focusing on sentence correctness. Together, they offer a multi-dimensional evaluation from linguistic fluency and grammatical correctness, ensuring texts meet basic language standards. Recognizing the complementary nature of perplexity and the CoLA classifier in capturing distinct aspects of model performance, we employ both to ensure a comprehensive evaluation. Specifically, we utilize KenLM [79], a language model, as our primary tool to calculate perplexity. We fine-tune two distinct KenLM models on both source style sentences and target style sentences. Subsequently, we use the refined target-style model to assess the perplexity of the generated sentence

Finally, to comprehensively evaluate the overall performance across all three key characteristics, we compute the geometric mean (denoted as Joint) of ACC, BLEU, and $\frac{1}{logPPL}$. When multiple references are available, we use multi-BLEU; otherwise, we select r-BLEU as the BLEU score for the calculation of the Joint score. The current equal-weight Joint score metric, while widely adopted in TST studies, leaves room for improvement regarding optimal weighting strategies.

#### 3.3.2. Human Evaluation

We collect human evaluation for the style strength, content preservation, fluency, and overall performance of the generated sentences. To achieve this, we invited three annotators who are fluent English speakers (each holding at least CET-6 certification or equivalent) and possess at least a master’s degree. Most of them have research experience in fields related to natural language processing. They assign scores on a scale from 1 to 5 for each aspect. A total of 50 samples from each algorithm are randomly selected and presented to the evaluators, resulting in 7300 annotated samples, with each sample receiving four scores. Many existing works do not provide details of the scoring standards, making it difficult to reproduce human evaluation. To address this, we have established specific scoring criteria for each of the three evaluation aspects. Raters strictly adhere to the standards while scoring, as follows:

Our evaluation considers each sentence’s text style strength, content preservation, text fluency, and overall quality using a scale of 1–5. Evaluators are simultaneously provided with the source style sentence, the reference sentence, and the candidates generated by different algorithms. For evaluating style strength, a fully transformed sentence into the target style scores 5, partial transformation scores 3, and no change in style scores 1. For content preservation, if the meaning is the same but expressed differently, it scores 3, and if the meaning is entirely different, it scores 1. For fluency, a sentence with no grammatical errors scores 5, minor grammatical errors that do not affect semantic expression score 3, and incoherent sentences score 1. As for the overall score, experts evaluate overall performance considering text style strength, content preservation, and text fluency. While experts usually use arithmetic averaging for overall scoring, there is a special case. When the candidate sentence is identical to the original, the text style strength score will be low, but the content preservation and text fluency scores will be high, resulting in a high overall score for the three indicators. In such cases, since no style correction has been made, we apply a penalty by multiplying the overall score by 0.6 to determine the final score. Inter-rater agreement is computed using Fleiss’ kappa coefficients across all evaluation dimensions: κ=0.68 for style accuracy, κ=0.63 for content preservation, and κ=0.71 for fluency. Detailed guidelines and the scoring results are available in our code repository.

### 3.4. Codebase

**Figure 1:**
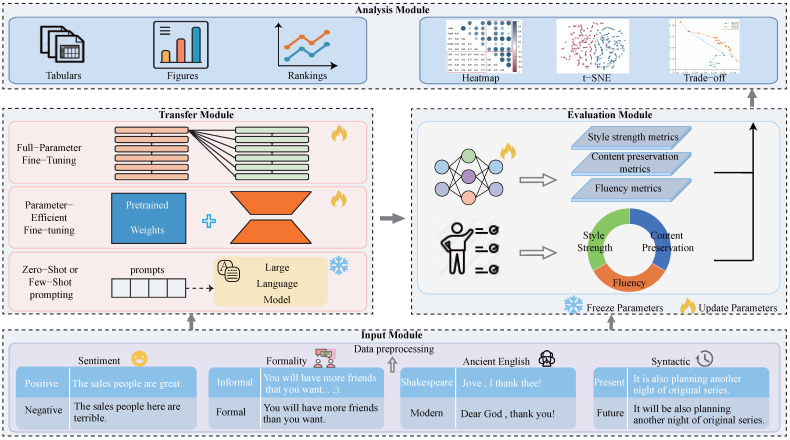
The modular based codebase of TSTBench. It consists of four modules, including input module, transfer module, evaluation module, and analysis module.

As shown in Figure 1, we design TSTBench following a structured framework. It consists of four modules, including the input module, transfer module, evaluation module, and analysis module. We outline each of the modules in more detail as follows:

**(1) Input module.** The input module comprises two steps: dataset pre-processing and prompt formulation. We provide text cleaning scripts to pre-process all input data, aiming to avoid incomplete pre-processing issues that could compromise the quality of text outputs. After this process, we prepend natural language to the testing sentence for the PEFT and ZSFS algorithms. More details will be presented in Appendix A.3.

**(2) Transfer module.** As depicted in Table 4, three sub-modules are provided corresponding to the three major TST methods (FPFT, PEFT, and ZSFS), which generate target-styled sentences.

**(3) Evaluation module.** This module includes style strength (measured by ACC using a RoBERTa classifier), content preservation (evaluated through comparisons of the output sentences with the input sentences using s-BLEU and s-BERT or with reference sentences using r-BLEU, r-BERT, and, if multiple references exist, multi-BLEU and multi-BERT), fluency (assessed by PPL and COLA), and overall score evaluation metrics (Joint score).

**(4) Analysis module.** We provide analysis tools such as heatmaps, t-SNE, and trade-off figures. Heatmaps assist in analyzing the correlation between evaluation metrics and human ratings, while t-SNE helps visualize the distribution of source-styled sentences and target-styled sentences. We have also introduced a standardized protocol for invoking the aforementioned functional modules to ensure fair and replicable TST evaluations, including various stages such as data pre-processing, style transfer, result evaluation, and analysis.

## 4. Evaluations and Analysis

In this section, we first describe the evaluation settings before presenting our findings. We start by giving an overview of model performance across various styles. Following this, we compare large language models (LLMs) with fine-tuned models in the context of text style transfer (TST). Finally, we examine the correlation between automatic evaluation metrics and human evaluations.

### 4.1. Experimental Settings

The installation packages and versions for each replication algorithm are available on our homepage. We utilized the API interface to evaluate the capability of LLMs. Specifically, the ChatGPT version tested in the experiment was “gpt-3.5-turbo-1106”, and the GPT-4 version was “gpt-4-0125-preview”. Additionally, for PEFT algorithms, we employed the 7B-parameter Llama [82] and quantized it to 4-bits using QLoRA [83] to fit our GPU memory.

Classifier models for evaluating style strength are trained on the Roberta-base model for three epochs to prevent overfitting. Evaluation based on BERTScore was conducted using the original implementation (https://github.com/Tiiiger/bert_score (accessed on 28 April 2025)). For perplexity evaluation, we utilize the KenLM (https://github.com/kpu/kenlm (accessed on 28 April 2025)) model trained on each transfer style. In evaluating COLA score, we employ the RoBERTa-large classifier trained on the CoLA corpus (https://huggingface.co/cointegrated/roberta-large-cola-krishna2020 (accessed on 28 April 2025)). Consistent hyper-parameter settings are maintained for various style transfer directions within the same dataset. The replication experiments for TSTBench were conducted on 1 Tesla A100 GPUs (40 GB).

Regarding hyper-parameter settings, if specific values are provided in the original paper, we replicate the experiments utilizing those values. Otherwise, we conduct grid searches within a reasonable range for hyper-parameters that yield optimal performance. In addition to replicating experiments from papers, we also aim to explore the generalization of classic algorithms across different datasets. Most papers only experiment on one or two datasets, but we strive to cover as many datasets as possible. We have supplemented experiments with algorithms on other styles, except for the following cases: (1) papers explicitly stating that they can only perform certain specific style tasks, (2) algorithms themselves having requirements regarding the size of the dataset, and (3) algorithms only applicable to parallel datasets.

### 4.2. Overview of Model Performances Across Styles

**Figure 2:**
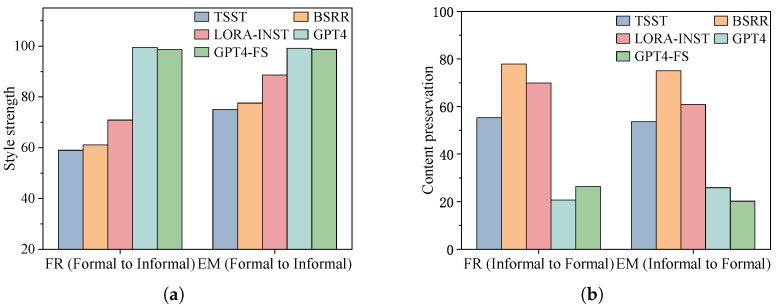
Comparison of style strength and content preservation performance across models on the GYAFC-EM and GYAFC-FR datasets. Subfigure (**a**) presents the comparison of style strength among models in the formality transfer task, while subfigure (**b**) depicts the comparison of content preservation.

Through the demonstration of Table A4, Table A5, Table A6, Table A7, Table A8, Table A9, Table A10, Table A11, Table A12, Table A13, Table A14 and Table A15 in Appendix B, we assess the performance of different models within every transfer direction. In order to visually discern the differences between these models, we represent the style strength, content preservation, and text fluency using bar graphs on the same dataset and style transformation direction, as depicted in Figure 2. Our findings indicate that no model excels in all evaluation metrics, nor are there universally applicable algorithms. TYB demonstrates superior generalization, achieving the highest joint scores in 6 out of 12 transfer directions. For more rigorous statistical validation, we calculate 95% confidence intervals for all Joint score comparisons to strengthen our findings. Our evaluation was conducted on four datasets that meet TYB’s requirement for parallel training data in supervised learning, with each dataset containing at least 1000 instances to ensure reliable statistical analysis. As shown in Table A16, there are statistically significant differences in performance between the models. TYB consistently shows superior performance across multiple datasets, with its confidence intervals not overlapping with those of other models. However, its reliance on parallel data for fine-tuning pre-trained models inherently limits its applicability in unsupervised scenarios (e.g., sentiment transfer). To address this limitation, future work could explore data augmentation techniques such as back-translation to generate pseudo-parallel data, thereby extending TYB’s utility to non-parallel settings. Following closely behind are the BSRR and TSST algorithms. The TSST algorithm, an unsupervised approach, even outperforms supervised methods in sentiment transfer. It employs transductive learning, where retrieved samples provide style examples in specific contexts. This enables the model to learn styles that match the content, thereby avoiding the generalization errors associated with inductive learning and achieving excellent performance. However, TSST faces challenges such as high time complexity in the retrieval process and a relatively complex model structure. Meanwhile, BSRR combines bootstrapping with reinforcement rewards, enabling efficient style transfer with minimal data. Despite its advantages, its performance relies on the data quality, which potentially limits its effectiveness in scenarios with data noise, such as the Amazon dataset.

Nonetheless, unsupervised algorithms consistently performed below average on COLA values. This suggests that unsupervised fine-tuning algorithms generated sentences that often do not adhere to grammatical rules. Despite this, the PPL evaluation metric performed well. Therefore, solely relying on PPL as a singular metric for evaluating semantic fluency is not justifiable; it requires a combined assessment with COLA values. Our observation further indicates that model performance varies across different transfer directions within the same dataset. For instance, on the GYFAC (FR) dataset, the TYB model performs exceptionally well in the informal to formal direction, while its performance in the formal to informal direction falls only at an average level. Consequently, it is crucial to specify the style direction when conducting TST evaluations in the future.

**Finding 1:** No single model performs exceptionally well across all evaluation metrics, and there are no universally applicable algorithms. Furthermore, as performance varies across different transfer directions within the same style, we recommend clarifying the direction of style transfer when evaluating its performance.

### 4.3. Comparison Between LLMs and Fine-Tuned Models on TST

**Figure 3:**
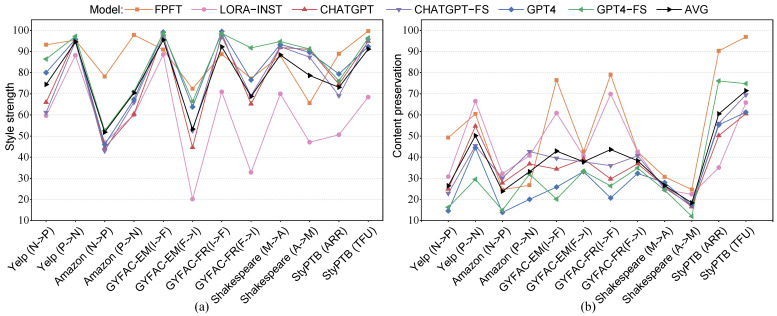
Performance comparison of models across text style transfer datasets. Subfigures (**a**) and (**b**) present style transfer strength and content preservation, respectively. The black line indicates the mean performance across all models, and for the line of full-parameter fine-tuning (FPFT), it represents the top-performing model in this category. (N → P) indicates negative-to-positive transfer, with (P → N) being the reverse; (I → F) denotes informal-to-formal, and (F → I) the reverse; (M → A) refers to modern-to-ancient English, with (A → M) as the reverse.

To better contextualize the performance of LLMs against fine-tuned models (including models of full-parameter fine-tuning (FPFT) and parameter-efficient fine-tuning (PEFT)), we selected the SOTA fine-tuned models for comparison with LLMs. Figure 3 illustrates the performance of ACC and BLEU scores across all datasets. Specifically, we aim to answer three main questions: (1) How do the capabilities of LLMs on TST compare to those of fine-tuned SOTA models in terms of automatic metrics? (2) How does an LLM’s performance on TST compare to that of a fine-tuned SOTA model in terms of human evaluators? (3) Do the current evaluation metrics align with the assessment of LLMs? Below, we present a detailed analysis.

**Figure 4:**
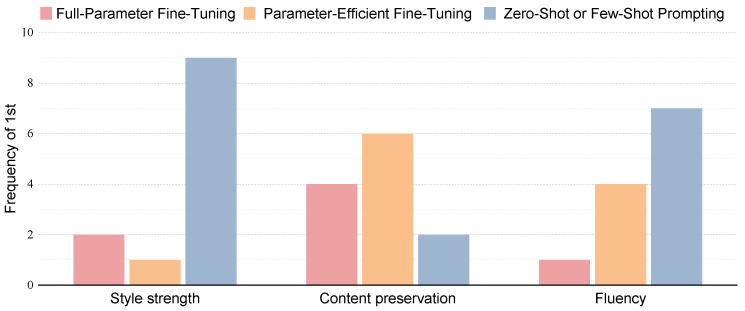
Based on human raters, the frequency of full-parameter fine-tuning, parameter-efficient fine-tuning, and zero-shot or few-shot prompting is selected to determine the optimal approach across various transfer styles, evaluated in terms of style strength, content preservation, and fluency, respectively.

(1) Overall, the performance of most LLMs does not surpass that of fine-tuned SOTA models. We identified multiple reasons that could contribute to such a circumstance. Firstly, LLMs tend to paraphrase input sentences, resulting in lower content preservation. Secondly, the linguistic expression of LLMs is more flexible, causing the distribution of generated text to be inconsistent with the input text. This leads to a less favorable performance when using string-based methods to measure the fluency of generated text compared to fine-tuned SOTA models. Therefore, even while 5 out of 12 datasets show superior performance by LLMs in terms of style strength, their advantage in the overall performance across three evaluation aspects is not significant. Moreover, LLMs exhibit disparities in performance compared to fine-tuned SOTA models, especially in understanding negative texts, where the sentiment polarity often remains unchanged during style transfer. Additionally, GPT4 demonstrates a better understanding of negative sentiment compared to ChatGPT, consistent with findings in references [84,85]. Furthermore, LLMs struggle to effectively remove adjectives and adverbs, tending to generate sentences with more adjectives and adverbs compared to humans, resulting in relatively unsatisfactory results when tasked with their removal.

(2) Human preference strongly favors LLMs in TST tasks, indicating the superior performance of LLMs compared to fine-tuned SOTA models across various datasets, as shown in Figure 4. Analysis using correlation heatmaps reveals that human ratings exhibit a marked preference for linguistic fluency, consistently awarding higher scores to semantically fluent text. LLMs demonstrate an adeptness at generating linguistically fluent text, particularly in terms of grammatical correctness. Notably, across all datasets, the highest COLA values are associated with text generated by LLMs. Moreover, human evaluations consider GPT4 to be the best model on one third of the test datasets.

(3) The automatic evaluation metrics designed for fine-tuned models may not be suitable for evaluating LLM models. As previously mentioned, evaluation metrics used for LLMs indicate significantly lower scores compared to those used for SOTA models, particularly in terms of content preservation as measured by BLEU and fluency as measured by PPL. Due to LLMs’ tendency to rewrite input sentences, string-based content preservation evaluation methods are also not applicable to LLMs, similarly affecting string-based metrics such as METEOR [86] and chrF [87]. Moreover, PPL is sensitive to the length of the text, displaying instability, especially for short texts [88]. Table 5 presents statistics on the generated sentence lengths, revealing that LLMs’ generated sentences are 17.75% longer for formal transfer and 23.1% longer for positive transfer than the average ground-truth text.

### 4.4. Human Correlation of the Evaluation Metrics

**Figure 5:**
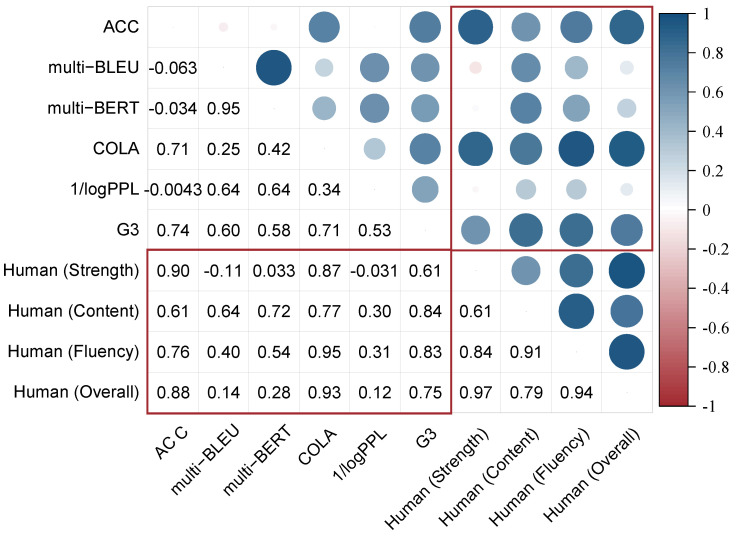
The Pearson correlation coefficient between human evaluations and automatic evaluation metrics. The bordered area indicates the relationships between human evaluations and automatic metrics. The size of the circles represents the magnitude of correlation, with blue indicating positive correlation and red indicating negative correlation.

To analyze the correlation between human evaluation metrics and automatic evaluation metrics, we adopt the Pearson correlation coefficient (PCC) for conducting a correlation analysis of the evaluation metrics. The obtained heatmap of evaluation metric correlation across different datasets is depicted in Figure 5. With values ranging from −1 to 1, the larger the absolute value of the number, the higher the correlation between the evaluation metrics. By presenting a heatmap for each typical style, the evaluation results can be clearly visualized and compared across different styles. Below, we present detailed results and their analyses.

(1) The correlation between the ACC classifier and human evaluations on sentiment transfer is limited due to the adversarial challenges arising within classification accuracy. When utilizing non-parallel sentiment datasets such as Yelp and Amazon, which contain human-generated sentences as ground truth references, lower accuracy results were surprisingly revealed when the ground truth was input into the accuracy (ACC) classifier during the testing process for the Amazon dataset and Yelp (Negative→Positive). As shown in Table 4, the ACC accuracy values are 64.8 and 45.6, respectively, which are notably lower than the average ACC classifier value of 81.7. This divergence can be attributed to the training data distribution disparities from the human-generated ground-truth data. Given that sentences following sentiment transfer occasionally differ by just a few words from those composed by humans, this similarity may mislead the classifier [89]. Therefore, in evaluating style intensity within sentiment transfer tasks, we suggest researchers consider the potential for adversarial examples resulting from similar word distributions that mislead classifier evaluations.

**Finding 2:** The disparity between human preference, which strongly favors LLMs in TST tasks, and the inadequacy of automatic evaluations suggests that current evaluation metrics may not be suitable for assessing the performance of LLM models. The performance of most LLMs does not exceed that of fine-tuned SOTA models.

**Figure 6:**
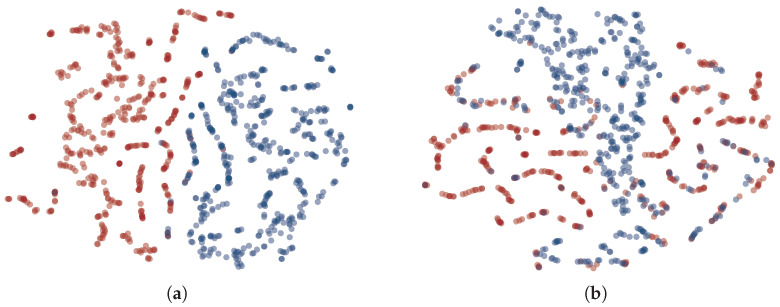
Visualization of the embedding space for two distinct styles using t-SNE [90] on test and reference data. The “CLS” embedding from the final layer of the classification model was used for this visualization. Subfigure (**a**) illustrates the test data space, while subfigure (**b**) shows the reference data space. Blue and red markers denote sentences with positive and negative style, respectively.

We utilized t-SNE [90] to visualize the embedding space of two distinct styles on test data and reference data. This was performed using the “CLS” embedding on the final layer of the classification model. The results of the testing and reference data spaces are depicted in Figure 6a and Figure 6b, respectively. These results indicate that the reference data are more intermixed, suggesting the difficulty in classifying the reference data using the classification model, which aligns with the aforementioned classifier accuracy results.

(2) The correlation between the evaluation metrics of content preservation and human evaluations varies depending on the dataset. The averaged PCC values for human evaluations of content preservation with BLEU and BERTScore values are 0.708 and 0.798, respectively, demonstrating a strong association and indicating consistency between the BLEU and BERTScore evaluation metrics and human assessment standards. Furthermore, regarding formality and sentiment styles, the correlation coefficient for the BERTScore is greater than that of BLEU. However, in modern English style transfer and fine-grained syntactic/semantic transfer, the PCC values for BLEU are higher than that of the BERTScore. One possible reason is that the BERT model has limited capability in understanding various syntactic expressions in ancient English. Therefore, it would be more reasonable to adopt different content preservation evaluation metrics for different style transfer tasks.

(3) The correlation between perplexity (PPL) and human ratings is not significant. A lower PPL score does not consistently reflect language that is similar to that used by humans [91]. Furthermore, the PCC for human ratings is 60.1% lower for PPL compared to COLA. Consequently, COLA proves to be more suitable for evaluating text fluency than PPL. Even in cases involving style shifts from modern to classical texts, where grammar rules differ, COLA can still maintain a high PCC value.

(4) The current automatic metrics struggle to provide a comprehensive evaluation. Although automatic evaluation metrics are highly correlated with human evaluation, the exact rankings do not align. Taking formality transfer on the GYAFC-FR dataset as an example, the order of the top five rankings by human is as follows: GPT4-ZS, CHATGPT-FS, CHATGPT-ZS, GPT4-FS, and TYB. However, the automatic evaluation rankings are as follows: TYB, LlaMa-LORA, LlaMa-LORA-INST, BSRR, and STRAP. The inconsistency in rankings indicates that the current evaluation metrics are insufficient. We recommend designing specific prompts, such as using chains of thoughts, to guide ChatGPT in evaluating the performance of different models.

**Finding 3:** The current automatic evaluation metrics are constrained by adversarial challenges, dataset-dependent variations in correlation coefficients, and the unreliability of perplexity scores compared to human ratings. Furthermore, disparities in the rankings of automatic evaluation metrics against human assessments highlight the need for specific prompts or guiding mechanisms to improve the robustness and applicability of these metrics.

### 4.5. Contents in Appendixes A and B

To maintain clarity and focus in the main body of our work, we have chosen to include several important elements in the Appendix. Below is a concise overview of the Appendix contents to guide readers in easily locating specific information:

- Appendix A Algorithms and implemented details in TSTBench:
  - Appendix A.1: Descriptions of algorithms in TSTBench;
  - Appendix A.2: Various evaluation methods in existing TST algorithms;
  - Appendix A.3: Prompts in our experiment.
- Appendix B Additional results and analysis:
  - Appendix B.1: Sentiment transfer;
  - Appendix B.3: Ancient English transfer;
  - Appendix B.4: Fine-grained syntactic and semantic style transfer;
  - Appendix B.5: Analysis of trade-off curves;
  - Appendix B.6: Case study.

## 5. Discussion

TSTBench is presented as a benchmarking platform for text style transfer that not only includes the implementation of a wide range of transfer models but also encompasses a set of metrics for assessing the quality of the generated texts. We expect that this new benchmark will contribute to the TST community in several key aspects: providing a clear overview of current advancements based on transformer in TST, including those involving large language models (LLMs); improving the reproducibility and reliability of future research; enabling researchers to effortlessly compare new methods with existing ones; and sparking new research inquiries through comprehensive evaluations.

The continuous advancement of sophisticated language models presents promising opportunities for future benchmark development. Notably, integrating evaluation paradigms such as LLM-as-a-judge [92] could harness these models’ growing capabilities to conduct more effective evaluations. Additionally, few-shot prompting techniques hold potential for more efficient quality assessment, reducing reliance on costly human evaluations.

Despite its strengths, TSTBench has certain limitations that encourage further investigation. We implement evaluation approaches as a baseline reference, recognizing that more sophisticated evaluation metrics are urgently needed for development, such as weighting schemes in the overall score. These observations open up promising avenues for future research within this domain.

## Figures and Tables

**Table 1:** Comparison with existing benchmarks in the text style transfer. These benchmarks mainly cover one or two domains and lack thorough analysis across different kinds of algorithms. Our TSTBench addresses this gap by providing a broader scope, encompassing multiple domains and offering in-depth analysis across various algorithms. “GYAFC” refers to GYAFC-related benchmark and “CDS” refers to CDS-related benchmark.

Benchmark	Style				Models						Evaluation				
					**FPFT**			**PEFT**	**ZSFS**		**Automatic Metrics**				**Human**
	**Sentiment**	**Formality**	**Syntactic/** **Semantic**	**Authorship**	**Embedding-** **Based**	**Decoder-** **Based**	**Classifier-** **Based**		**Zero-** **Shot**	**Few-** **Shot**	**Strength**	**Content**	**Fluency**	**Overall**	
GYAFC [10]		✓			✓	✓					✓	✓	✓	✓	✓
MATST [11]	✓			✓	✓	✓					✓	✓	✓		
CDS [9]				✓	✓	✓					✓	✓	✓	✓	✓
StylePTB [12]			✓		✓	✓						✓			
XFORMAL [13]		✓			✓	✓					✓	✓	✓	✓	✓
LMStyle [14]	✓	✓							✓		✓	✓	✓		
**TSTBench (Ours)**	✓	✓	✓	✓	✓	✓	✓	✓	✓	✓	✓	✓	✓	✓	✓

**Table 2:** The categorization of algorithms in TSTBench, according to their type of tuning, their strategy of controlling text style, and their implementation methodology.

Type	Strategy	Method	Algorithm
Full-parameter fine-tuning (FPFT)	Embedding-based	Conditional GAN	STYTRANS [4]
		Transductive learning	TSST [6]
		Style-related words deleting	NAST [50]
	Decoder-based	Paraphrase generation	STRAP [9]
		Reinforcement learning	BSRR [8], TYB [7]
	Classifier-based	Edit entangled latent representation	CTAT [5]
Parameter-efficient fine-tuning (PEFT)	On task dataset	LoRA-based fine-tuning	LlaMa-LORA [62]
	On instruction dataset	LoRA-based instruction-tuning	LlaMa-LORA-INST [62]
Zero-shot or few-shot prompt tuning (ZSFS)	Zero-shot	Large language model	CHATGPT [63], GPT4 [66]
	Few-shot	Large language model	CHATGPT-FS [63], GPT4-FS [66]

**Table 3:** Dataset details in TSTBench, where the words in blue signify the source style, and the words in red indicate the target style. We evaluate our benchmark on 7 commonly used datasets (Yelp [36], Amazon [71], GYAFC-EM [10], GYAFC-FR [10], Shakespearet [72], StylePTB-TFU [12], and StylePTB-ARR [12]).

Dataset	Style	Train	Valid	Test	Examples
Yelp	Negative	180K	2000	500	She wasn’t happy being there.
	Positive	270K	2000	500	She seemed happy to be there.
Amazon	Negative	277K	1015	500	I don’t see what others have liked about this.
	Positive	278K	985	500	I see exactly what others have liked about this.
GYAFC-EM	Informal	52,595	2877	1416	Different from what i’ve seen though.
	Formal	52,595	2356	1082	It differs from what i have seen, however.
GYAFC-FR	Informal	51,967	2788	1332	So if you ’re set on that, that ’s the way to go!!
	Formal	51,967	2247	1019	If you are set on that, that is the way to go.
Shakespeare	Ancient	18,395	1218	1462	And art thou changed?
	Modern	18,395	1218	1462	And now you’ve changed?
StylePTB-TFU	Present	4377	243	243	The dividend had been five cents a share.
	Future	4377	243	243	The dividend will have been five cents a share.
StylePTB-ARR	Origin	6544	364	364	Third they offer high yields.
	Removal	6544	364	364	Third they offer yields.

**Table 4:** We evaluated the style strength of the ground truth text using the Roberta and TextCNN classifiers, respectively, and evaluated its grammatical acceptability using the Cola classifier. In this table, higher accuracy in evaluating the ground truth indicates better performance of the respective evaluation model. (N → P) indicates negative-to-positive transfer, with (P → N) being the reverse; (I → F) denotes informal-to-formal, and (F → I) the reverse; (M → A) refers to modern-to-ancient English, with (A → M) as the reverse.

Dataset	Roberta	TextCNN	COLA	Dataset	Roberta	TextCNN	COLA
Yelp (N → P)	64.8	62.2	90.2	GYAFC-FR (I → F)	90.2	89.5	96.5
Yelp (P → N)	95.4	89.8	86.2	GYAFC-FR (F → I)	86.7	82.3	87.8
Amazon (N → P)	45.6	39	85.8	Shakespeare (A → M)	92.7	80.3	94
Amazon (P → N)	61.8	55.4	85	Shakespeare (M → A)	85.7	85.2	63.8
GYAFC-EM (I → F)	90.4	89.1	93.1	StylePTB-ARR	82.7	80.5	36.2
GYAFC-EM (F → I)	85	83.9	82.8	StylePTB-TFU	99.7	96.8	58.5

**Table 5:** Statistics of sentence lengths generated by different models. Reference denotes the average length of ground-truth sentences in the test set of the dataset, while fine-tuned models represents the mean sentence length generated by both full-parameter fine-tuning and parameter-efficient fine-tuning methods. The last four columns show the sentence lengths generated by GPT-series models.

Dataset	Reference	Fine-Tuned Models	CHATGPT	CHATGPT-FS	GPT4	GPT4-FS
YELP (N->P)	8.48	8.7	9.83	10.03	10.62	10.37
YELP (P->N)	8.25	8.6	9.55	10.18	9.12	10.15
AMAZON (N->P)	9.86	10.3	11.61	11.68	13.14	13.18
AMAZON (P->N)	10.57	11.1	12.02	11.86	12.54	11.72
GYAFC_EM (I->F)	10.87	11.8	12.4	12.01	12.84	13.68
GYAFC_EM (F->I)	10.21	10.2	10.56	10.73	10.36	10.12
GYAFC_FR (I->F)	11.15	12.7	13.14	12.9	13.45	13.33
GYAFC_FR (F->I)	10.72	10.7	10.78	10.29	10.4	9.26
SHAKESPEARE (A->M)	8.47	9.12	9.3	9.49	9.49	9.75
SHAKESPEARE (M->A)	8.94	9.2	9.52	9.41	9.34	9.7
STYLEPTB_ARR	8.03	7.9	7.31	7.41	6.93	7.88
STYLEPTB_TFU	10.38	10.3	10.19	10.29	10.35	10.4

**Figure A1:**
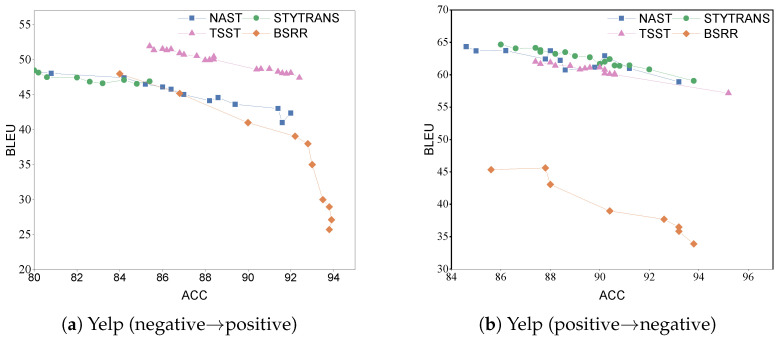
Trade-off analysis on Yelp: (**a**) Negative-to-positive transformation; (**b**) Positive-to-negative transformation.

## Data Availability

Data will be made available on request.

## Appendix A. Algorithms and Implemented Details in TSTBench

### Appendix A.1. Descriptions of TST Algorithms

**STYTRANS** [4] employs a discriminator to maximize the probability of the generated sentences matching the desired style without disentangling latent representations of the input sentences, and it leverages the capabilities of the attention mechanism within the Transformer to improve both style transfer and content preservation.

**TSST** [6] utilizes an attentional encoder–decoder framework within a retriever setup. This method incorporates the top-K relevant sentences in the target style during the transfer process. In this approach, a context-aware style embedding is leveraged to mitigate the inconsistency problem.

**NAST** [50] is an edit-based method that locates style-related words through discrete operations (typically referring to word deletion, retrieval, and generation). This method offers good interpretability. However, when complex structural changes are required in sentences, relying solely on these few discrete word-level operations may not achieve optimal results.

**STRAP** [72] generates pseudo-parallel data by feeding sentences from various styles into a diverse paraphrase model serving as a tool for style regularization. Then, another paraphrase model is trained to revert these paraphrased sentences back to their original style, which is used to perform style transfer during the inference stage.

**BSRR** [8] and **TYB** [7] use the reinforcement learning approach to design a reward function for fine-tuning the policy network, providing control conditions as feedback rewards for pre-trained model fine-tuning. Specifically, through a well-trained style discriminator, normalized attention scores are obtained and used to determine the reward values for each word in the generated sentences, i.e., step-by-step rewards, which are utilized as fine-grained signals for policy gradient back-propagation.

**CTAT** [5] treats the task of text style transfer as an optimization problem, with the model divided into three parts: an encoder $E_{\theta_{e}}$ that encodes the input text *x* into the latent space *z*, a decoder that decodes *z* to obtain $x^{\prime }$, and a classifier $C_{\theta_{c}}$ that classifies the text style. In this optimization problem, the objective function aims to find $z^{\prime }$ closest to *z*, resulting in $x^{\prime }$ with the desired style $y^{\prime }$, subject to the constraint that the classifier identifies it as the target style, as expressed in the formula below.

(A1) $$z^{\prime }=\min_{z^{*}}∥z^{*}-E_{\theta_{e}}\left(x\right)∥\;s.t.\;C_{\theta_{c}}\left(z^{*}\right)=y^{\prime }.\;$$

The advantage of CTAT is its high flexibility; however, its downsides include the impact on generation speed as well as the need for further improvement in text fluency.

**LlaMa-LORA** [62] is an efficient fine-tuning approach that employs the low-rank adaptation method. LORA works by adding trainable low-rank matrix pairs (matrices A and B) alongside the frozen pre-trained weights, allowing updates to be represented as ΔW=AB, where the rank of A and B is significantly smaller than that of the original weight matrices. This method substantially reduces the number of trainable parameters while maintaining model performance, making the fine-tuning of large language models more efficient and feasible.

In our reproduction experiments, we implement LORA fine-tuning in two ways: (1) using task-specific parallel datasets to fine-tune Llama 7B [93], adapting it specifically for the requirements of the TST task; and (2) conducting LORA fine-tuning with the Alpaca instruction dataset (LlaMa-LORA-INST), which consists of 52,000 instruction samples [82]. Notably, instruction tuning enhances the capabilities and generalization of LLMs for our TST task, enabling effective performance on both parallel and non-parallel datasets.

**ChatGPT** [63] excels in conversational scenarios, generating text [94] that closely aligns with human language patterns through reinforcement learning and fine-tuning from human feedback. According to [94], by utilizing a single prompt that provides multiple examples of sentences being “rewritten” into a specific style, ChatGPT can extrapolate and rephrase text in unseen styles.

**GPT4** [66] is recognized as a more advanced generation model, outperforming previous LLMs. In this paper, we focus on exploring the performance of ChatGPT and GPT-4 in zero-shot and few-shot (*-FS) style transfer.

**Table A1:** Existing TST models utilize various transfer tasks, datasets, and evaluation metrics to assess their performance.

Model	Dataset	Style Strength	Content Preservation	Fluency	Overall
STYTRANS [4]	Yelp, Imdb	FastText	s_BLEU, r_BlEU	PPL (KenLM)	-
TSST [6]	Yelp, GYAFC-FR	BERT-based	s_BLEU, r_BLEU	PPL (KenLM)	G4 (s-BLEU, r-BLEU, ACC, Fluency)
NAST [50]	Yelp, GYAFC-FR	RoBERTa-base	s_BLEU, multi_BLEU	PPL (GPT2-base)	G2, H2 (multi-BLEU, ACC)
STRAP [9]	GYAFC-EM, Shakespeare	RoBERTa-large	SIMILE	COLA	J (ACC; Content; Fluency)
BSRR [8]	Yelp, Amazon, GYAFC-EM/FR	Roberta-base	multi_BLEU, multi_BERT	-	G2, H2 (multi-BLEU, ACC)
TYB [7]	GYAFC-EM/FR	TextCNN	BLEURT, multi_BLEU	-	H2 (multi-BLEU, ACC)
CTAT [5]	Yelp, Amazon	FastText	multi_BLEU	SRILM	-

### Appendix A.2. Various Evaluation Methods in Existing TST Algorithms

New methods are often inadequately evaluated with differing evaluation setups, which leads to the utilization of diverse evaluation metrics to assess the performance of model outputs, as shown in Table A1.

### Appendix A.3. Prompts in Our Experiment

We first utilized the following prompt [94]: “Here is a rewrite of the text, which is more positive”. However, we observed that in strongly emotional contexts, ChatGPT tended to diminish the negative sentiment of the sentence rather than transfer the sentiment into a positive one. To address this, we modified the prompt to “change the sentiment of the sentence from negative to positive”, and this adjustment effectively altered the sentiment of the sentence. Table A3 shows the prompts for few-shot learning and Table A2 shows the prompts for zero-shot learning, respectively. These exemplars are randomly selected from the training data to prevent overfitting and ensure fair comparison with other models.

**Table A2:** The list of prompts used in zero-shot experiments.

Dataset	Prompts
**Yelp**	Rewrite the following sentence, maintain the content and change the sentiment of the sentence from negative to positive:
	Rewrite the following sentence, maintain the content and change the sentiment of the sentence from positive to negative:
**Amazon**	Rewrite the following amazon comment, maintain the content and change the sentiment of the sentence from negative to positive:
	Rewrite the following amazon comment, maintain the content and change the sentiment of the sentence from positive to negative:
**GYAFC-EM/FR**	Change the style of the sentence from informal to formal:
	Change the style of the sentence from formal to informal:
**Shakespeare**	Change the style of the following sentence from Shakespeare English to modern English:
	Change the style of the following sentence from modern English to Shakespeare English:
**StylePTB-ARR**	Remove adjectives and adverbs of the following sentence:
**StylePTB-TFU**	Convert the following sentence into the future tense:

**Table A3:** The list of prompts used in few-shot experiments.

Dataset	Prompts in Few-Shot Experiments
**Yelp**	Here are some examples of how to rewrite: example 1. ’bottom line : overpriced, bad service, shitty beer .’ is rewritten as ’Bottom line: premium pricing, good service, and unique-tasting beer.’; example 2. ’this is the worst giant eagle i ’ve ever been to .’ is rewritten as ’this is the best giant eagle i ’ve ever been to .’; example 3. ’the constant passing of guests was quite irritating .’ is rewritten as ’The regular flow of guests brought a lively and engaging atmosphere.’\Now rewrite the sentence:
**Amazon**	Here are some examples of how to rewrite: example 1. ’the worst part is the grate … not sure what else to call it .’ is rewritten as ’The beat part is the grate—quite a unique element indeed.’; example 2. ’the color is much different in person than how it appears online .’ is rewritten as ’The color is even more captivating in person than it appears online!’; example 3. ’if you still want it, wait at least till the price drops dont pay $ for it . not reccommeneded .’ is rewritten as ’if you still want it, wait till the price drops. highly reccommeneded .’\Now rewrite the sentence:
**GYAFC-EM**	Here are some examples of how to change: example 1. ’the movie the in-laws not exactly a holiday movie but funny and good!’ is rewritted as ’the in-laws movie isn’t a holiday movie, but it’s okay.’; example 2. ’they are comming out with plenty more games.’ is rewritten as ’they are coming out with many more games.’; example 3. ’i dunno but i just saw the preview for the season finale and i am sooooooooooo excited!!!’ is rewritten as ’i don’t know, but i just saw the preview for the season finale. i’m sooooo excited!’\Now change the style of the sentence:
**GYAFC-FR**	Here are some examples of how to change: example 1. ’i need to know what 2 do’ is rewritten as ’i need to know what to do.’; example 2. ’sms if you still can’t contact, 5.’ is rewritten as ’message me and if you have any trouble contacting me, try the number five.’; example 3. ’i don’t know but if you find out please let me know lol’ is rewritten as ’i do not know but if you find out please inform me.’\Now change the style of the sentence:
**Shakespeare**	Here are some examples of how to change: example 1. ’i have a mind to strike thee ere thou speak’st .’ is rewritten as ’i have half a mind to hit you before you speak again.’; example 2. ’well, i know not what counts harsh fortune casts upon my face, but in my bosom shall she never come to make my heart her vassal.’ is rewritten as ’well, i can not tell how my difficult life has weathered my face, but i will never let those difficulties subdue my courage.’; example 3. ’my visor is philemon’s roof ; within the house is jove .’ is rewritten as ’my mask is like the roof of the poor’\Now change the style of the sentence:
**StylePTB-ARR**	Here are some examples of how to remove: example 1. ’we are having a regular day’ is rewritten as ’we are having a day’; example 2. ’the successful launch continues a unk recovery in the u.s. space-science program’ is rewritten as ’the launch continues a recovery in the u.s. space-science program’; example 3. ’in national over-the-counter trading unk shares fell num cents to num’ is rewritten as ’in trading unk shares fell num cents to num’\Now remove adjectives and adverbs of the sentence:
**StylePTB-TFU**	Here are some examples of how to convert: example 1. ’i ’m happy and unk he said’ is rewritten as ’i will be happy and unk he will say’; example 2. ’united illuminating ’s plan however offers more for unsecured creditors’ is rewritten as ’united illuminating ’s plan however will offer more for unsecured creditors’; example 3. ’those rights prevent anyone other than revco from unk a reorganization plan’ is rewritten as ’those rights will prevent anyone other than revco from unk a reorganization plan’\Now convert the sentence:

## Appendix B. Additional Results and Analysis

The performance of various models for each transfer direction is detailed in Table A4, Table A5, Table A6, Table A7, Table A8, Table A9, Table A10, Table A11, Table A12, Table A13, Table A14 and Table A15. Alongside these results, we provide an analysis categorized by style types.

### Appendix B.1. Sentiment Transfer

Table A4 presents the results for the style transfer from ”negative → positive” on Yelp dataset. Overall, as evident from the column Joint, full-parameter fine-tuning models (e.g., TSST) excel over inference-based LLMs (e.g., GPT-4) in three metrics defined in Section 3.3. Specifically, models such as TSST and BSRR exhibit higher accuracy scores, demonstrating their superiority in terms of style strength. However, LlaMa-LORA-INST performs better in BLEU and BERT-related evaluations, indicating its proficiency in comprehending sentiment style transfer instructions, thereby preserving content more effectively. As anticipated, LLMs are capable of generating more fluent texts, particularly in terms of grammatical correctness. Nevertheless, LLMs tend to rephrase texts, leading to poorer content preservation. Surprisingly, LLMs struggle to comprehend negative texts, resulting in the sentiment polarity remaining unchanged during the style transfer.

**Table A4:** Results on Yelp dataset (style transfer from negative to positive). The column **ACC**, columns **s-BLEU** to **multi-BERT**, **COLA** to **PPL**, and **Joint** represent style strength, content preservation, fluency, and overall performance, respectively. An upward-pointing arrow ↑ indicates that a higher value of this metric is preferable, while a downward-pointing arrow ↓ signifies that a lower value is better.

Model	ACC ↑	s-BLEU ↑	r-BLEU ↑	Multi-BLEU ↑	s-BERT ↑	r-BERT ↑	Multi-BERT ↑	COLA ↑	PPL ↓	Joint ↑
REFERENCE	64.8	33.9	100	95.6	91.4	100	91.3	90.2	101.6	11
STYTRANS	80.2	**61.8**	28.7	48.7	**94.4**	90.2	89.6	49	199.9	9
TSST	**93.2**	57.8	28.3	49.3	94.1	**90.4**	89.7	47.6	119.5	**9.9**
NAST	88.8	54	26.8	45.6	93.7	90	89.4	45	189.5	9.2
BSRR	92.2	56.5	21	39.1	94.2	89.8	89.1	77.8	**83.3**	9.3
CTAT	69.6	26.3	10.2	19.5	86.9	85	84.6	6	466.5	6
LlaMa-LORA-INST	59.6	51.5	**30.8**	**51.7**	92.5	89.6	**90**	94	109.5	8.7
CHATGPT-ZS	66	40.4	25	41	91.5	89.2	89.5	**96**	112	8.3
CHATGPT-FS	61.2	39.5	22.9	38.6	91.4	88.8	89.1	95.8	135.2	7.8
GPT4-ZS	80	22.3	14.6	23.3	89	87.6	87.7	95.6	206.1	7
GPT4-FS	86.4	24.6	16.3	25.2	89	87.6	87.8	95.6	220.8	7.4

**Table A5:** Results on Yelp dataset focusing on style transfer from positive to negative sentiment.

Model	ACC ↑	s-BLEU ↑	r-BLEU ↑	Multi-BLEU ↑	s-BERT ↑	r-BERT ↑	Multi-BERT ↑	COLA ↑	PPL ↓	Joint ↑
REFERENCE	95.4	31	100	91.5	91.6	100	92.1	86.2	75.3	12.6
STYTRANS	93.8	61.7	28.4	60.4	95.4	90.4	91.6	66	138.9	10.5
TSST	95.4	60.6	28.7	60.5	95.2	90.5	91.6	63	108.4	10.7
NAST	89.2	**66.6**	30.4	64.3	**96.2**	**90.9**	92.2	67.6	136.9	10.5
BSRR	98	44.8	20	42.8	92.4	89.8	90.2	94.8	**82.3**	9.8
CTAT	68.6	27.5	10.3	23.2	87.4	85.2	85.4	10.2	359.6	6.5
LlaMa-LORA-INST	88.2	59.4	**32.6**	**66.5**	94.7	90.2	**92.3**	92.6	108.1	**10.8**
CHATGPT-ZS	96	44.8	25.6	54.7	93.2	89.6	91.6	96.2	98.1	10.5
CHATGPT-FS	94.8	38.7	23	45.4	92	89	90.7	**97**	90.4	9.8
GPT4-ZS	95.8	37.4	22.8	44.5	92.3	89.1	90.8	96	142.1	9.5
GPT4-FS	**97.2**	27.7	15.9	29.5	90.3	87.6	89	96.4	186.9	8.2

Table A5 shows the results for the style transfer from ”positive → negative”. Similar patterns are observed. However, there is one exception. Both LlaMa-LORA-INST and LLMs exhibit a remarkable enhancement in accuracy, indicating that instruction fine-tuning and inference methods can comprehend positive texts more effectively than negative ones. As a result, LlaMa-LORA-INST attains the superior overall performance, while LLMs are comparable to full-parameter fine-tuning models in this task.

Table A6 and Table A7 show the results for the style transfer from “negative → positive” and “positive → negative”, respectively, on the Amazon dataset. The BSRR algorithm exhibited a significantly higher style intensity on the Amazon dataset compared to other models, surpassing similar models by 10–20 points. However, its content preservation performance was not satisfactory. Upon reviewing its generated results, we found that the algorithm utilized a shortcut, generating “XXX is worth it” when transforming from negative to positive, and “XXX is not worth it” in the reverse case. Here, XXX represents the subject of the input sentence, i.e., the object in an Amazon review. For example, when the input is “speedloader is very hard to use especially with an extended magazine”. the output is “this speedloader is worth it”. This shortcut ensures style transformation intensity while maintaining the input’s subject to balance some degree of content preservation. A potential reason could be that it applies reinforcement learning with token-level rewards, potentially leading the model to output words that yield higher reward values. Additionally, the basic policy gradient algorithm does not effectively balance the model’s exploration and exploitation capabilities. As a result, the training process may easily fall into locally optimal solutions.

In terms of content preservation, the algorithms STYTRANS, TSST, NAST, and LlaMa-LORA-INST all performed well. However, due to its high language flexibility, the LLM did not excel in BLEU score. Regarding style strength, GPT4 outperformed ChatGPT, particularly on the Yelp dataset, with a margin of 12–18 points. This indicates that GPT4 has a stronger understanding of negative sentiment compared to ChatGPT, consistent with the findings in references [84,85].

**Table A6:** Results on Amazon dataset focusing on style transfer from negative to positive sentiment.

Model	ACC ↑	s-BLEU ↑	r-BLEU ↑	s-BERT ↑	r-BERT ↑	COLA ↑	PPL ↓	Joint ↑
REFERENCE	45.6	47.1	100	92.8	100	85.8	267.5	9.3
STYTRANS	32.4	**83.3**	39.3	**97.3**	91.1	74.8	144.4	6.4
TSST	49.8	73.4	**39.4**	96.2	90.8	69	119.5	7.4
NAST	42	80.6	38.9	**97.3**	**91.2**	70.6	150.3	6.9
BSRR	**78.2**	41.9	24.6	89.9	87.5	94.8	**48.3**	**7.9**
CTAT	60	40.9	19.5	92.3	88.1	38	146.4	6.2
LlaMa-LORA-INST	47	48.4	32.2	92.1	90.1	94.8	213.1	6.6
CHATGPT-ZS	44.4	39.5	27.8	92.2	90.2	**95.8**	196.5	6.2
CHATGPT-FS	43	44	30.1	92.2	89.9	93.4	212.2	6.2
GPT4-ZS	46	18.3	13.9	89.4	88.1	**95.8**	327.2	4.8
GPT4-FS	52.6	20.4	14.9	89	87.8	**95.8**	456.2	5

**Table A7:** Results on Amazon dataset focusing on style transfer from positive to negative sentiment.

Model	ACC ↑	s-BLEU ↑	r-BLEU ↑	s-BERT ↑	r-BERT ↑	COLA ↑	PPL ↓	Joint ↑
REFERENCE	61.8	52.2	100	93.6	100	85	247.3	10.4
STYTRANS	34.8	**80.6**	44.2	96.8	91.6	65	165.1	6.7
TSST	49	80.2	**44.3**	**97.5**	**92.2**	74.2	113.2	7.7
NAST	51	74.7	40.6	96.6	91.5	64.2	165.6	7.4
BSRR	**97.8**	42.3	26.7	89.9	88	93	**32.3**	**9.1**
CTAT	52.4	48.5	26.1	93.6	89.5	32.8	200.8	6.4
LlaMa-LORA-INST	60	56.1	40.8	94.3	91.6	91.4	156.9	7.9
CHATGPT-ZS	60.4	49.7	36.8	93.8	91.5	93.8	124.7	7.7
CHATGPT-FS	66	57.5	42.7	94.7	92	91.4	140.2	8.3
GPT4-ZS	67.4	25.8	20.1	90.9	89.2	**95.4**	219.8	6.3
GPT4-FS	71	42.8	32	92.8	90.5	92.8	193.2	7.6

### Appendix B.2. Formality Transfer

Referring to the results in Table A8 and Table A10, it is evident that LLMs consistently maintain exceptionally high accuracy in the “informal to formal” transformation, showcasing significantly better style transfer strength than other models. The abundance of formal text in its pre-training corpus allows the LLM to leverage rich prior knowledge, resulting in the formalization and modification of most words in source-style sentences. However, this process leads to a substantial decrease in content preservation in the generated text. Notably, after integrating three example sentences as prompts, ChatGPT demonstrates improved content preservation in the generated text. Furthermore, fine-tuning LlaMa-LORA on the GYFAC_EM and GYAFC-FR datasets yields the best overall performance, excelling in both content preservation and semantic fluency, even surpassing ChatGPT. In contrast, algorithms fine-tuned using pre-trained language models such as STRAP, BSRR, and TYB exhibit very good fluency, indicating high-quality text generation from pre-trained models. But the content preservation of the STRAP model is poor, and a potential reason could be that when STRAP initially leverages a paraphrase model for normalization, it may lose some content information. Subsequently, when generating target-styled sentences through specific style models, there can be deviations in content. Through qualitative assessment, it was found that the output sentences may contain some content unrelated to the input sentence.

Upon reviewing Table A9 and Table A11, we notice a significant decrease in LLMs’ style transfer strength in the “formal to informal” scenario. Without fine-tuning on the task dataset, LLMs generate texts that substantially differ from the reference answers, limiting the evaluation of informal texts in this task. Even with the inclusion of three example prompts in a few-shot scenario, coverage of most cases in the test set remains insufficient. Fine-tuned models adapt better to the diverse nature of informal texts and, consequently, achieve superior overall performance by fitting the dataset distribution more effectively.

**Table A8:** Results on GYAFC-EM dataset focusing on style transfer from informal to formal.

Model	ACC ↑	s-BLEU ↑	r-BLEU ↑	Multi-BLEU ↑	s-BERT ↑	r-BERT ↑	Multi-BERT ↑	COLA ↑	PPL ↓	Joint ↑
REFERENCE	90.4	34.5	100	100	92.2	100	94.4	93.1	124.7	12.3
STYTRANS	57.3	45.4	22.4	40.9	91.5	89.1	89	38.9	411.1	7.3
TSST	75.1	55.2	29.3	53.6	93.4	91	90.9	59.4	121.6	9.4
NAST	71.9	61.5	31.7	53.5	94.4	91.3	91.3	48.6	258	8.8
STRAP	74.8	23.2	18.2	32.7	90.5	89.8	89.8	90.7	112.8	8
BSRR	77.6	**62.6**	43.4	75.1	94.4	92.4	92.3	87.9	111.2	10.7
TYB	90.9	58.7	44.3	76.5	**95**	**94.2**	**94**	93.5	105.4	**11.4**
CTAT	89.7	16.5	9	15.2	87.7	86.5	86.5	21.4	174.6	6.4
LlaMa-LORA-INST	88.6	40.3	35.8	60.9	91.9	91.5	91.4	93.7	124.8	10.4
LlaMa-LORA	76.6	60.2	**45.3**	**78.8**	93.9	92.7	92.6	91.6	**105.2**	10.9
CHATGPT	98	15.6	20.6	34.4	89.1	89.8	89.7	96.4	170.2	8.7
CHATGPT-FS	97.4	17.4	22.9	39.5	89.4	90.2	90.1	**96.8**	156.7	9.1
GPT4	**99.2**	10.1	15.3	25.9	88.1	89.1	89	96.7	270	7.7
GPT4-FS	98.7	8.2	11.8	20.2	87.3	88.2	88.2	96	345.6	7

**Table A9:** Results on GYAFC-EM dataset focusing on style transfer from formal to informal.

Model	ACC ↑	s-BLEU ↑	r-BLEU ↑	Multi-BLEU ↑	s-BERT ↑	r-BERT ↑	Multi-BERT ↑	COLA ↑	PPL ↓	Joint ↑
REFERENCE	85	19.4	100	100	89.9	100	91	82.8	197	11.7
STYTRANS	59.7	65.3	16.4	30.8	94.8	88.2	87.5	60.3	254.3	6.9
TSST	**74.6**	57.3	16.6	32.7	94.2	88.1	87.4	56.7	209.1	7.7
NAST	74.5	60.2	16.5	28.6	94.7	88.4	87.7	59.1	315.1	7.2
STRAP	47.3	24.4	12.2	21.7	90.8	87.2	86.8	91.6	270.1	5.7
BSRR	72.4	**60.8**	22.1	42.7	**95.5**	89.3	88.6	85.9	324.2	**8.1**
TYB	44.6	48.5	26.1	49.1	95.2	**90.8**	**89.8**	90.4	141.4	7.6
CTAT	61.5	30.4	6.7	12.4	89.5	85	84.6	18.3	328.9	5.1
LlaMa-LORA-INST	20.2	56.3	22.6	40.6	94.6	89.2	88.6	95.2	191	5.4
LlaMa-LORA	36.9	57.3	**27.5**	**48**	94.7	90	89.1	91.2	**134.3**	7.1
CHATGPT-ZS	44.6	33.5	20.5	39.2	92.4	89	88.6	96.3	138.7	7.1
CHATGPT-FS	52.6	31.2	19.7	37.8	91.9	88.8	88.3	**96.7**	150.2	7.3
GPT4-ZS	63.8	22.5	16.9	33.2	90.8	88.4	88	95.7	173.6	7.4
GPT4-FS	66.4	22.3	16.8	33.4	90.6	88.3	88	95.4	172.9	7.6

**Table A10:** Results on GYAFC-FR dataset focusing on style transfer from informal to formal.

Model	ACC ↑	s-BLEU ↑	r-BLEU ↑	Multi-BLEU ↑	s-BERT ↑	r-BERT ↑	Multi-BERT ↑	COLA ↑	PPL ↓	Joint ↑
REFERENCE	90.2	35.6	100	100	92.4	100	94.4	96.5	67.2	12.9
STYTRANS	68.5	48.1	26.3	43.9	91.7	89.6	89.5	32.1	318.7	8.1
TSST	59	60.6	31.6	55.3	92.5	89.8	89.7	41	109	7.7
NAST	56.8	**71.5**	37.2	61.1	96	92.1	92	63.4	137.3	8.9
STRAP	78.2	23.5	21.3	38.4	90.8	90.4	90.4	95.4	**48**	9.2
BSRR	61.1	66.9	47	77.9	95	92.5	92.4	90.7	66.2	10.4
TYB	88.7	56.7	47.5	79	**95.1**	**94.3**	**94.2**	97	52.3	**12.1**
CTAT	90.3	21.1	12.5	20.5	88.8	87.5	87.4	27.6	100.3	7.4
LlaMa-LORA-INST	70.9	52.9	42	69.9	93.3	91.9	91.9	95.7	71.8	10.5
LlaMa-LORA	63.7	63.4	**48.6**	**81.5**	94.5	92.9	92.8	95.5	59.4	10.8
CHATGPT-ZS	98.5	12.2	17.3	29.7	88.4	89	89	**98.9**	115.5	8.5
CHATGPT-FS	97.1	15.4	21	36	89.1	89.6	89.6	98.6	98.9	9.1
GPT4-ZS	**99.5**	7	11.9	20.7	87.4	88.2	88.2	98.3	195.3	7.3
GPT4-FS	98.6	9.8	15.2	26.4	88.3	88.9	88.9	98.9	164.3	8

**Table A11:** Results on GYAFC-FR dataset focusing on style transfer from formal to informal.

Model	ACC ↑	s-BLEU ↑	r-BLEU ↑	Multi-BLEU ↑	s-BERT ↑	r-BERT ↑	Multi-BERT ↑	COLA ↑	PPL ↓	Joint ↑
REFERENCE	86.7	20.4	100	100	90	100	90.4	87.8	106.2	12.3
STYTRANS	79.9	70	18.2	32.4	95.2	88.2	87	62.7	166.6	8
TSST	91.1	50.1	15.8	31.6	91.7	86.8	85.8	46.6	128.8	8.4
NAST	66.1	**70.4**	19.6	32.6	95.9	88.9	87.6	68.9	149.3	7.6
STRAP	33.2	23.5	12.9	25.7	91.8	88.1	87.1	95.5	**61.4**	5.9
BSRR	77.2	67.4	23.1	42.6	**96.2**	89.3	88.1	92.4	170.9	8.6
TYB	49.2	54.5	27.9	47.7	96	**90.9**	**89.3**	96.8	69.9	8.2
CTAT	82.4	29.6	8.9	15	89.5	85.5	84.7	28.2	135.9	6.3
LlaMa-LORA-INST	32.8	53.7	23.1	42.4	94.3	89.2	88.2	98.3	91.4	6.8
LlaMa-LORA	53.8	53.6	**29.4**	**51.5**	94.3	90.2	88.8	96.2	70.2	**8.7**
CHATGPT-ZS	65.2	28.4	19.4	36.9	91.7	88.7	87.9	**99.4**	78.7	8.2
CHATGPT-FS	69.1	32.1	21.4	40.7	92.1	88.9	88	98.7	89.1	8.6
GPT4-ZS	76.6	18.4	16.3	32.3	90.4	88.1	87.4	98.9	94.7	8.2
GPT4-FS	**91.7**	20.7	16.2	34.9	90.5	88	87.4	94.2	146.8	8.6

### Appendix B.3. Ancient English Transfer

Table A12 and Table A13 show that both ChatGPT and GPT4 outperform LlaMa-LORA fine-tuned on the task and instruction datasets based on Llama, presenting excellent performance in both transfer directions. This could be attributed to the models’ pre-training data containing the works of Shakespeare, and LLMs having more domain-specific knowledge. GPT4 also shows an improvement in overall scores compared to ChatGPT. Overall, the performance of transforming modern text into ancient text is not as good as that of transforming ancient text into modern text. This may be due to differences in semantics, syntax, and expression habits between modern and ancient English. It is important to note that in this specific task, the perplexity (PPL) value does not effectively indicate poor fluency. Human evaluation in this task also has significant limitations because it is not easy to find professionals who are knowledgeable in ancient English. The replicability and reference value of manual scoring criteria in this task are very limited.

**Table A12:** Results on Shakespeare dataset focusing on style transfer from ancient to modern languages.

Model	ACC ↑	s-BLEU ↑	r-BLEU ↑	s-BERT ↑	r-BERT ↑	COLA ↑	PPL ↓	Joint ↑
REFERENCE	92.7	22.1	100	90	100	94	137.7	12.3
STYTRANS	38.3	65	20.6	94.9	89.5	40.1	263.5	5.2
TSST	60.5	45.1	19.7	91.9	88.8	36.7	144.1	6.2
NAST	31.1	55.5	18.3	93.7	89.1	35.8	377	4.6
STRAP	84.3	16.9	14.2	89.7	88.8	88.2	**91**	6.4
BSRR	56.6	**68.8**	27.3	**95.9**	90.7	69.7	207.9	6.6
TYB	88.1	39.5	30.7	92.9	91.4	90.8	118	**8.3**
CTAT	45.1	3.7	2	83.5	83.2	10.5	177.9	2.6
LlaMa-LORA-INST	70	53	24.6	90.8	88.3	80.6	227.7	6.8
LlaMa-LORA	72.2	54.9	**31.3**	94.1	**91.5**	85	177.9	7.6
CHATGPT-ZS	91.9	25.6	26.3	88.5	88.9	94.7	169	7.8
CHATGPT-FS	92.3	24.6	25.4	88.4	88.8	94.5	169.6	7.7
GPT4-ZS	93.4	26.2	28	88.9	89.2	94	181	8
GPT4-FS	**94.7**	18.6	24.4	88	88.8	**96.2**	176.3	7.6

**Table A13:** Results on Shakespeare dataset focusing on style transfer from modern to ancient langauges.

Model	ACC ↑	s-BLEU ↑	r-BLEU ↑	s-BERT ↑	r-BERT ↑	COLA ↑	PPL ↓	Joint ↑
REFERENCE	85.7	22	100	90	100	63.8	226.8	11.6
STYTRANS	86.3	53.8	15.9	91.7	87.2	40.1	274.8	6.3
TSST	87.3	46.2	17.3	91.9	88	52.6	130.7	**6.8**
NAST	63.3	61	18.5	**94.6**	88.7	59.9	303.7	5.9
STRAP	61.1	17.1	8.8	90	87.4	79.6	**125.7**	4.8
BSRR	85.6	61.2	17.8	94.4	89	65.1	346.1	6.4
TYB	65.6	51.2	24.7	94.4	90.5	85.4	160.1	**6.8**
CTAT	**95.3**	5	2	84	82.7	10	200.6	3.3
LlaMa-LORA-INST	47.1	**70.9**	22.5	92.8	87.7	**89.9**	235.2	5.8
LlaMa-LORA	55	47.4	**25.4**	94.3	**90.6**	86.7	162.5	6.5
CHATGPT-ZS	91	35.5	16.6	88.9	85.9	88.7	339.6	6.4
CHATGPT-FS	87.3	32.8	17.3	88.8	86.2	89.3	306.4	6.4
GPT4-ZS	89.6	23.3	17.3	88	86.4	83.5	355.8	6.4
GPT4-FS	91.2	13.1	12	86.4	85.4	81.2	419.1	5.7

### Appendix B.4. Fine-Grained Syntactic and Semantic Style Transfer

From Table A14 and Table A15, we observe that algorithms fine-tuned on the task dataset (BSRR, TYB, and LlaMa-LORA) exhibit superior performance compared to pre-trained models without fine-tuning. These fine-tuned algorithms demonstrate enhanced style transfer strength, content preservation, and fluency. Conversely, instruction fine-tuning on LlaMa-LORA yields inferior results compared to other algorithms in this context, possibly due to a limited understanding of the command, with no discernible advantage in fine-grained style transfer through command comprehension. GPTs struggle to effectively identify adjectives and adverbs, often leading to unnecessary sentence rephrasing and, consequently, reduced semantic preservation. GPT4 demonstrates higher accuracy compared to ChatGPT and shows better overall performance. The GPT models tend to generate sentences with more adjectives and adverbs compared to a human, so its performance is relatively unsatisfactory when tasked with removing adjectives and adverbs. It is worth noting that some sentences in this dataset lack components and contain many unknown words (’unk’). During transformations, the language model predicts or removes ’unk’ to ensure the completeness of generated sentences, resulting in lower BLEU scores. Nevertheless, this should not necessarily be interpreted as indicative of poor model performance.

Both ChatGPT and GPT-4 tend to generate complete and coherent sentences. However, in some sentences within the dataset, if adjectives and adverbs are removed without other modifications, it may lead to sentences that lack coherence due to missing components, further affecting GPT’s ability to remove adjectives and adverbs. Specific scores also indicate that the LLAMA model, fine-tuned through targeted LlaMa-LORA, achieves better performance in this task. Additionally, GPT-4 and ChatGPT score higher than other algorithms in the CoLA metric, which evaluates sentence grammar.

**Table A14:** Results on Styleptb-ARR dataset, which focuses on style transfer by removing adjectives and adverbs from the text.

Model	ACC ↑	s-BLEU ↑	r-BLEU ↑	s-BERT ↑	r-BERT ↑	COLA ↑	PPL ↓	Joint ↑
REFERENCE	82.7	65.5	100	96.9	100	36.2	124.6	12
BSRR	88.9	**64.9**	**90.3**	**96.8**	**99**	35.8	123	**11.9**
TYB	**91.8**	60.8	88.9	96.4	98.9	36.6	124.8	**11.9**
LlaMa-LORA-INST	50.6	38.8	35.1	91.3	90.7	61.3	796.6	6.4
LlaMa-LORA	88.5	62.3	89.3	96.5	98.9	36.2	**122.8**	11.8
CHATGPT-ZS	74.5	43.6	50.2	92.9	92.8	**38.3**	419.3	8.5
CHATGPT-FS	69.1	48.7	56	94	94	43.2	350	8.7
GPT4-ZS	79.4	44	55.3	92.9	93.4	44	425	9
GPT4-FS	76.1	61.4	76.1	96	97	40.3	169.6	10.4

**Table A15:** Results on Styleptb-TFU dataset, which involves style transfer from the present tense to the future tense.

Model	ACC ↑	s-BLEU ↑	r-BLEU ↑	s-BERT ↑	r-BERT ↑	COLA ↑	PPL ↓	Joint ↑
REFERENCE	99.7	55.9	100	97.3	100	58.5	70.7	13.3
BSRR	99.2	56.6	96.6	**97.4**	**99.7**	56	69.4	13.1
TYB	**99.7**	55.7	**96.9**	97.3	**99.7**	58.2	**68.7**	**13.2**
LlaMa-LORA-INST	68.4	55.8	65.8	95.6	96.1	63.7	237.4	9.4
LlaMa-LORA	95.9	**57.1**	90.3	**97.4**	99.3	58.8	76.7	12.6
CHATGPT-ZS	94.8	37	60.6	93.9	95.4	**78.8**	230.5	10.2
CHATGPT-FS	95.3	43.4	69.5	94.8	96.4	73.6	168.7	10.9
GPT4-ZS	92.3	39.1	61.3	94.2	95.6	76.4	233.3	10.1
GPT4-FS	96.4	48.2	74.8	96.2	97.6	61.8	152.2	11.3

**Table A16:** Shown are the 95% confidence intervals (CI) for Joint score comparisons across four typical datasets. GYAFC-EM (I → F) refers to informal-to-formal transfer, GYAFC-EM (F → I) to the reverse, Shakespeare (M → A) to modern-to-ancient English, and Shakespeare (A → M) to the reverse.

Model	GYAFC-EM (I → F)	GYAFC-EM (F → I)	Shakespeare (M → A)	Shakespeare (A → M)
BSRR	(10.625, 10.775)	(8.052, 8.148)	(6.553, 6.647)	(6.367, 6.433)
TYB	(11.337, 11.463)	(7.555, 7.645)	(8.257, 8.343)	(6.761, 6.839)
LORA-INST	(10.324, 10.476)	(5.351, 5.449)	(6.763, 6.837)	(5.76, 5.84)
LORA	(10.824, 10.976)	(7.047, 7.153)	(7.56, 7.64)	(6.464, 6.536)
CHATGPT	(8.638, 8.762)	(7.044, 7.156)	(7.754, 7.846)	(6.357, 6.443)
CHATGPT-FS	(9.028, 9.172)	(7.25, 7.35)	(7.652, 7.748)	(6.366, 6.434)
GPT4	(7.642, 7.758)	(7.355, 7.445)	(7.959, 8.041)	(6.358, 6.442)
GPT4-FS	(6.935, 7.065)	(7.554, 7.646)	(7.56, 7.64)	(5.669, 5.731)

### Appendix B.5. Analysis of Trade-Off Curves

Researchers in [36,50] point out the need to evaluate the trade-off curve to assess the balance between algorithm performance in style strength and content preservation. We provide the trade-off curves in our benchmark to demonstrate this trade-off relationship, as shown in Figure A1.

### Appendix B.6. Case Study

In order to gain a deeper understanding of the characteristics of various models, several sentences are extracted from each dataset and assessed across different models. The resulting outputs are presented in Table A17, Table A18, Table A19, Table A20, Table A21, Table A22, Table A23, Table A24, Table A25, Table A26, Table A27 and Table A28, which provides us with valuable insights for intuitively analyzing the quality of generated text. Taking dataset GYAFC-EM dataset as an example, it is observed that the FPFT models can rewrite one word for informal expressions, but it is challenging to completely rewrite all informal expressions within a single sentence. Even TYB and BSRR models are unable to accomplish this. Among PEFT models, LlaMa-LORA performs well in fine-tuning on task datasets by identifying and correctly rewriting all three instances that needed to be modified. However, LlaMa-LORA-INST fine-tuning on the instruction dataset still cannot recognize all informal expressions. In contrast, LLM can identify all instances of informal expressions and provide corresponding rewrites. The text generated by GPT-4 is longer, with more extensive revisions, even to the point of being redundant. This leads to higher accuracy but also results in more changes to the original sentence, longer generated sentences, and a decrease in BLEU scores compared to other models.

**Table A17:** Examples generated by models on the yelp dataset from negative to positive.

	From Negative to Positive
Original text	Since their visit, the only scorpions we’ve seen were dead !
STYTRANS	Since their visit, the only scorpions we’ve seen best popular !
TSST	Since their visit, the best scorpions we’ve seen were delicious !
NAST	Since their visit, the best scorpions we’ve eaten were best !
BSRR	The only scorpions we’ve seen were dead and it is good.
CTAT	Since their visit, the only delicious we arrowhead disappointment has were dining !
LlaMa-LORA-INST	Since their visit, the only scorpions we’ve seen were alive!
CHATGPT	Since their visit, we have seen live scorpions!
CHATGPT-FS	Since their visit, we have only seen live scorpions!
GPT4	Since their visit, we haven’t seen any scorpions alive!
GPT4-FS	Since their visit, we’ve only encountered peaceful, non-threatening scorpions!

**Table A18:** Examples generated by models on the Yelp dataset from positive to negative.

	From Positive to Negative
Original text	The wait staff is extremely attractive and friendly !
STYTRANS	The wait staff is extremely attractive and rude !
TSST	The wait staff is extremely arrogant and rude !
NAST	The wait staff is extremely attractive and rude !
BSRR	The wait staff is extremely attractive but it is not good.
CTAT	Wait the service is these pressure and telling family wasn’t that customer.
LlaMa-LORA-INST	The wait staff is extremely unattractive and unfriendly!
CHATGPT	The wait staff is not at all attractive and unfriendly.
CHATGPT-FS	The wait staff is unattractive and unfriendly.
GPT4	The wait staff is quite unattractive and unfriendly!
GPT4-FS	The wait staff is quite off-putting and superficial!

**Table A19:** Examples generated by models on the Amazon dataset from negative to positive.

	From Negative to Positive
Original text	I purchased this jawbone hd as a replacement for an older jawbone thinker.
STYTRANS	I purchased this jawbone hd as a replacement for an older jawbone turquoise.
TSST	I purchased this jawbone hd as a replacement for an older jawbone capacity.
NAST	I purchased this jawbone hd as a replacement for an older jawbone thinker.
BSRR	I purchased this jawbone hd as a replacement for an older jawbone thinker and this was worth it.
CTAT	I purchased this jawbone icon as a replacement for an older iphone num_extend useful piece.
LlaMa-LORA-INST	I purchased this jawbone hd to replace my older jawbone thinker and i’m so glad i did!
CHATGPT	I purchased this Jawbone HD as an upgrade for my older Jawbone Thinker.
CHATGPT-FS	I purchased this Jawbone HD as an upgrade for my older Jawbone Thinker.
GPT4	I bought this Jawbone HD to upgrade from an older Jawbone model, and I’m thrilled with the improvement!
GPT4-FS	I purchased this Jawbone HD as an upgrade from an older Jawbone Thinker.

**Table A20:** Examples generated by models on the Amazon dataset from positive to negative.

	From Positive to Negative
Original text	Then i thought something was wrong with my car.
STYTRANS	Then i thought something was wrong with my car.
TSST	Then i thought something was wrong with my car.
NAST	Then i thought something was wrong with my car.
BSRR	I thought something was wrong with my car and this was not worth it.
CTAT	Then i thought something was wrong with my car . then not green months.
LlaMa-LORA-INST	Then i thought something must be wrong with my car.
CHATGPT	I then thought something was wrong with my car.
CHATGPT-FS	then i thought something was right with my car.
GPT4	Then I realized something must be wrong with my car.
GPT4-FS	Then I was convinced there was nothing wrong with my car.

**Table A21:** Examples generated by models on the GYAFC-EM dataset from informal to formal.

	From Informal to Formal
Original Text	It doesn’t make sense …wait oooh I get it, still not funny.
STYTRANS	It does not make sense.
TSST	It does not make sense. wait, I get not it, still not funny.
NAST	It does not make sense. wait excuse I get it, still not funny.
STRAP	It does not make any sense, wait for me to finish.
BSRR	It does not make sense. wait, I get it. still not funny.
TYB	It doesn’t make sense. wait, I get it. still not funny.
CTAT	It doesn’t make sense. i still get it funny, however.
LlaMa-LORA-INST	It does not make sense…wait, I get it, still not funny.
LlaMa-LORA	It does not make sense. I get it, but it is still not funny.
CHATGPT	It is not logical…oh, I understand now, but it is still not humorous.
CHATGPT-FS	It is not comprehensible. Oh, I understand now. It is still not humorous.
GPT4	It does not make sense…Ah, I understand now; nevertheless, it remains unfunny.
GPT4-FS	The logic initially eluded me; however, upon further reflection, I comprehend the intent, yet it fails to amuse.

**Table A22:** Examples generated by models on the GYAFC-EM dataset from formal to informal.

	From Formal to Informal
Original text	Plus, for an older man, that is really attractive.
STYTRANS	Plus, for an older man, that is really .. !
TSST	Plus, for an older man, that ’s really sexy.
NAST	Plus, for an older man, that is really hot !
STRAP	Plus, it apos;s really cool for an older guy.
BSRR	Plus for an older man that is really hot
TYB	Plus, for an old man, that is really hot.
CTAT	Plus, for an older man that is really, attractive than he’s not that
LlaMa-LORA-INST	Plus, for an older man, that’s really attractive.
LlaMa-LORA	Plus, for an older man, that is really hot!
CHATGPT	And, like, for an older dude, that’s super hot.
CHATGPT-FS	And he’s really attractive for an older guy.
GPT4	Plus, for an older guy, that’s super attractive.
GPT4-FS	Also, for an older guy, that’s super attractive.

**Table A23:** Examples generated by models on the GYAFC-FR dataset from informal to formal.

	From Informal to Formal
Original text	Sounds strange but it worked in about 3–5 days for me!
STYTRANS	Sounds strange but it worked in about circumstance days for me pornographic.
TSST	It is but it worked in about five days for me!
NAST	sounds strange but it worked in about 3–5 days for me.
STRAP	I have it weird, but it works for me about three-five days a week!
BSRR	It sounds strange, but it worked in about three-5 days for me.
TYB	It sounds strange, but it worked in about three-five days for me.
CTAT	It sounds strange but in the situation is about me, i had feelings for num days.
LlaMa-LORA-INST	It sounds strange, but it worked in about three to five days for me.
LlaMa-LORA	It sounds strange, but it worked in about three to five days for me.
CHATGPT	It may seem unusual, but it proved effective for me within a period of 3–5 days.
CHATGPT-FS	It may sound unusual, but it proved effective within a period of approximately three to five days for me.
GPT4	It may seem unusual, but it proved effective for me within a period of three to five days.
GPT4-FS	It may seem unusual, but it proved effective for me within a period of three to five days.

**Table A24:** Examples generated by models on the GYAFC-FR dataset from formal to informal.

	From Formal to Informal
Original text	Stay very far away from this man.
STYTRANS	Stay very far away from this man stay far
TSST	Stay very far away from this guy
NAST	Stay very far away from this man !
STRAP	Dont come near him.
BSRR	Stay very far away from this guy
TYB	Stay very far away from this guy.
CTAT	Stay away from very far u have this guy and remember my best friend thats nothing ?
LlaMa-LORA-INST	Stay as far away from this guy as possible.
LlaMa-LORA	Stay very far away from this guy.
CHATGPT	Keep your distance from that guy.
CHATGPT-FS	Stay way far from this dude.
GPT4	Steer clear of this dude, seriously.
GPT4-FS	Keep your distance from this dude.

**Table A25:** Examples generated by models on the Shakespeare dataset from ancient English to modern English.

	From Ancient English to Modern English
Original text	I stretch it out for that word “broad, ” which, added to the goose, proves thee far and wide a broad goose.
STYTRANS	I stretch it out for that word ” broad, ” which, added to the goose, proves out far and wide a broad goose.
TSST	I swear it out for that’s ” really, ” which, has to the same, of you far and wide a its eye.
NAST	I stretch it out for that word ” broad on ” just on added to the goose on proves you far and wide a broad goose.
STRAP	I’ll stretch the word “broad” to mean “wide, wide, ” which, added to the goose, will prove you a wide goose.
BSRR	I’m stretching it out for that word “broad, ” which, added to the goose, proves you far and wide a broad
TYB	I stretch it out for that word “broad, ” which makes you far and wide a broad goose.
CTAT	I know it out that which, “quoth i, ” to part the candle itself, and a big piece of good opinion, “
LlaMa-LORA-INST	I stretch it out for that word “broad,” which, added to the goose, proves you to be far and wide a broad goose.
LlaMa-LORA	I stretch it out for that word “broad, ”which, added to the goose, proves you to be far and wide a broad goose.
CHATGPT	I emphasize the word “broad,” which, when added to the goose, proves that you are a wide and broad goose.
CHATGPT-FS	I extend it for the word “broad,” which, when added to the goose, proves you to be a very foolish person.
GPT4	I extend it for that word “broad,” which, when associated with the goose, clearly shows you are widely known as a complete fool.
GPT4-FS	I emphasize the term “broad,” which, when associated with the goose, clearly labels you as a complete fool everywhere.

**Table A26:** Examples generated by models on the Shakespeare dataset from modern English to ancient English.

	From from Modern English to Ancient English
Original text	Why, isn’t all this joking better than groaning about love ?
STYTRANS	Wherefore, is not all this joking better than groaning hither love ?
TSST	Why, is not all this ’t better than thy of love ?
NAST	Why, is not all this joking better than groaning of love ?
STRAP	Why do we laugh more than we weep ?
BSRR	Why, hath not all this joking better than groaning about love ?
TYB	Why, is not all this jesting better than groaning ?
CTAT	Why, is it possible that ever unk from all the gods, have some achieve thee for thy love and drown her permission.
LlaMa-LORA-INST	Why, is not all this joking better than groaning about love?
LlaMa-LORA	Why, is not this jesting better than groaning ?
CHATGPT	Wherefore, is not all this jesting finer than lamenting o’er love?
CHATGPT-FS	Wherefore, is not all this jesting finer than lamenting o’er love?
GPT4	Why, doth not all this jesting prove more sweet than to lament on love’s harsh sting?
GPT4-FS	Why, doth not this jesting prove a better sport than lamenting ’pon love’s woes?

**Table A27:** Examples generated by models on the STYLEPTB-ARR dataset.

	Remove Adjectives and Adverbs
Original text	There are still others who don’t mind seeing new unk unk.
BSRR	There are others who don’t mind seeing unk unk.
TYB	There are others who don’t mind seeing unk.
LlaMa-LORA-INST	There are still others who don’t mind seeing new.
LlaMa-LORA	There are others who don’t mind seeing unk.
CHATGPT	There are others who do seeing new unk unk.
CHATGPT-FS	There are still others who don’t mind seeing new unk unk.
GPT4	There are others who don’t mind seeing new.
GPT4-FS	There are still others who don’t mind seeing unk

**Table A28:** Examples generated by models on the STYLEPTB-TFU dataset.

	Convert into the Future Tense
Original text	I unk i was buying a cd as a cd he says.
BSRR	I will unk i will be buying a cd as a cd he will say.
TYB	I will unk i will be buying a cd as a cd he will say.
LlaMa-LORA-INST	I will be buying a cd as a cd he says.
LlaMa-LORA	I unk i will be buying a cd as a cd he will say.
CHATGPT	I will be buying a cd as a cd he will say.
CHATGPT-FS	I will think i will be buying a cd as a cd he will say.
GPT4	I will think i will be buying a cd, as he will say.
GPT4-FS	I will unk i will be buying a cd as a cd he will say.
